# Supplementary material for: Extreme optical nonlinearities unveiled by ultrafast laser filamentation in semiconductors
Source: Nat Commun. 2026 Feb 14;17:1701. doi: 10.1038/s41467-026-69530-w (PMC12909925; doi:10.1038/s41467-026-69530-w)
Supplement: Supplementary file 1 — Supplementary Information [file 41467_2026_69530_MOESM1_ESM.pdf]

Supplementary Information

Extreme optical nonlinearities unveiled by ultrafast laser filamentation in semiconductors

Maxime Chambonneau *[1], Markus Blothe [1], Vladimir Yu. Fedorov [2], Isaure de Kernier [3], Stelios Tzortzakis [4,5], and Stefan Nolte [1,6]

[1]Friedrich Schiller University Jena, Institute of Applied Physics, Abbe Center of Photonics, Albert-Einstein-Straße 15, 07745 Jena, Germany
[2]Laboratoire Hubert Curien, Université Jean Monnet, Saint-Etienne, France
[3]First Light Imaging S.A.S., Europarc Ste Victoire Bât. 5, Route de Valbrillant, 13590 Meyreuil, France
[4]Institute of Electronic Structure and Laser (IESL), Foundation for Research and Technology–Hellas (FORTH), P.O. Box 1527, GR-71110 Heraklion, Greece
[5]Materials Science and Engineering Department, University of Crete, 71003 Heraklion, Greece
[6]Fraunhofer Institute for Applied Optics and Precision Engineering IOF, Center of Excellence in Photonics, Albert-Einstein-Straße 7, 07745 Jena, Germany
* maxime.chambonneau@uni-jena.de

Contents

Supplementary Note 1	Optical properties		1
Supplementary Note 2	Metrology		1
	2.1	Nonlinear propagation imaging technique	1
	2.2	Necessary imaging conditions	2
	2.3	Absence of cumulative effects	3
	2.4	Repeatability	3
Supplementary Note 3	Linear propagation regime		3
	3.1	Linear propagation calculations	3
	3.2	Maximum fluence in the linear regime	5
	3.3	Group velocity dispersion	5
	3.4	Chirped temporal profiles	6
Supplementary Note 4	Filamentation regime		6
	4.1	Propagation morphology	6
	4.2	Energy profiles	7
	4.3	Critical power for nonlinearities	7
	4.4	Multi-photon absorption coefficient	10
	4.5	Summary tables	11
	4.6	Applications beyond laser direct writing	12

Supplementary Note 1 Optical properties

As a state of the art for linear and nonlinear refraction in various solids, the band gap E_g, the linear and nonlinear refractive indices (n_0 and n_2, respectively) and the corresponding references are given in Supplementary Table 1 for a selection of 30 materials. In the Primary Manuscript, Fig. 1, the relation $n_2 \propto E_g^{-3}$ is verified (red dashed curve).

Supplementary Table 1 Band gap E_g, linear, and nonlinear refractive indices (n_0 and n_2, respectively) of 30 materials. The data are given at 300 K, and at a wavelength of $\lambda = 1960$ nm or close to. For birefringent materials, n_0 is averaged over the ordinary and extraordinary refractive indices.

Material	E_g (eV)		n_0		n_2 (m^2/W)	
Ge	0.66	[S1]	4.11	[S2]	1.20×10^{-17}	[S3, S4]
Si	1.11	[S1]	3.45	[S2]	8.71×10^{-18}	[S5–S11]
InP	1.34	[S12]	3.13	[S13]	2.57×10^{-17}	[S10, S14–S17]
GaAs	1.43	[S1]	3.34	[S18]	1.31×10^{-17}	[S10, S11, S19, S20]
AMTIR-1 ($Ge_{33}As_{12}Se_{55}$)	1.81	[S21]	2.68	[S22]	3.38×10^{-18}	[S10]
ZGP ($ZnGeP_2$)	2.00	[S23]	3.17	[S24]	6.55×10^{-18}	[S11]
AMTIR-6 (As_2S_3)	2.08	[S25]	2.43	[S26]	2.50×10^{-18}	[S10]
GaSe	2.10	[S27]	2.58	[S28]	2.14×10^{-18}	[S11]
GaP	2.25	[S29]	3.04	[S30]	1.80×10^{-18}	[S31]
ZnTe	2.26	[S32]	2.72	[S33]	3.10×10^{-18}	[S11]
$CdSiP_2$	2.45	[S34]	3.06	[S35]	1.00×10^{-18}	[S36]
KRS-5 (TlBr-TlI)	2.50	[S37]	2.40	[S38]	2.60×10^{-18}	[S11]
AGS ($AgGaS_2$)	2.65	[S39]	2.39	[S40]	2.31×10^{-18}	[S11]
ZnSe	2.70	[S41]	2.44	[S42]	1.50×10^{-18}	[S10]
BGSe ($BaGa_4Se_7$)	2.73	[S43]	2.46	[S44]	1.96×10^{-18}	[S11]
KRS-6 (TlBr-TlCl)	3.25	[S37]	2.21	[S45]	1.21×10^{-18}	[S11]
4H-SiC	3.27	[S46]	2.58	[S47]	8.60×10^{-19}	[S48]
GaN	3.40	[S41]	2.30	[S49]	9.02×10^{-19}	[S50]
ZnS	3.60	[S41]	2.27	[S51]	5.50×10^{-19}	[S10]
LGS ($LiGaS_2$)	3.93	[S52]	2.05	[S53]	5.70×10^{-19}	[S11]
Si_3N_4	5.20	[S54]	1.98	[S55]	2.40×10^{-19}	[S56]
KTA ($KTiOAsO_4$)	5.24	[S57]	1.79	[S58]	1.75×10^{-19}	[S11]
Diamond	5.48	[S46]	2.38	[S59]	8.20×10^{-20}	[S60]
AlN	6.20	[S46]	2.13	[S61]	2.30×10^{-19}	[S62]
Sapphire (Al_2O_3)	8.30	[S46]	1.74	[S63]	2.44×10^{-20}	[S64]
Fused silica (a-SiO_2)	9.00	[S65]	1.44	[S66]	2.80×10^{-20}	[S10]
BaF_2	10.6	[S67]	1.46	[S68]	3.20×10^{-20}	[S10]
LiF	10.9	[S69]	1.38	[S70]	9.80×10^{-21}	[S10]
CaF_2	11.8	[S67]	1.42	[S68]	1.70×10^{-20}	[S10]
MgF_2	12.4	[S71]	1.37	[S68]	1.10×10^{-20}	[S10]

Concentrating more specifically on the semiconductors investigated in this study, the nonlinear refraction and absorption coefficients are summarized in Supplementary Table 2.

Supplementary Table 2 Nonlinear refraction and absorption coefficients for the investigated semiconductors at a wavelength of $\lambda = 1960$ nm or close to. Here, P_{cr}, β_2, and β_3 are the critical power for self-focusing, the 2- and 3-photon absorption coefficient, respectively. The critical power is calculated as $P_{cr} = \alpha\lambda^2/(4\pi n_0 n_2)$, where $\alpha = 1.8962$ for Gaussian beams [S72], and n_0 as well as n_2 are given in Table 1.

**Material**	P_{cr} (kW)	β_2 (m/W)	β_3 (m^3/W^2)	Reference
Ge	12.2	7.10×10^{-9}	–	[S4, S73–S75]
Si	20.1	1.96×10^{-12}	–	[S5, S6, S76]
InP	7.5	–	1.41×10^{-25}	[S77, S78]
GaAs	13.8	–	3.84×10^{-25}	[S20, S77–S80]

Supplementary Note 2 Metrology

2.1 Nonlinear propagation imaging technique

We detail here the experimental method used throughout the study, namely nonlinear propagation imaging. The experimental setup is schematically depicted in Supplementary Fig. 1(a). It relies on ultrashort laser pulses focused with an objective lens (Mitutoyo, M Plan Apo NIR 20×, NA = 0.40) onto the exit surface of the sample. The focusing lens is mounted on a precision linear translation stage (Physik Instrumente, M-404.1DG) to finely adjust the focal position along the optical axis z.

The beam is then imaged using an inverted infrared microscope working in transmission, which comprises an imaging objective (Olympus LCPLN100XIR, NA = 0.85), a tube lens (Thorlabs TTL200-S8), and an extended InGaAs camera (First Light Imaging, C-RED 2 ER 2.2 μm [S81]). After magnification, the calibrated field of view is 87.4×70.0 μm^2, and the corresponding pixel size is 137 nm. Neutral density filters are inserted in the detection path to maintain a high dynamic range and avoid camera saturation or damage. The transmission of these filters is measured over the input spectrum prior to the experiment.

The imaging lens position is fixed to image the exit surface of the sample under white light illumination, ensuring that no plasma is generated in air between the imaging objective and the sample, which would otherwise degrade image quality. The procedure to reconstruct 3D quantitative fluence distributions is as follows:

1. A very low input pulse energy E_{in} (typically a few pJ) is used to ensure linear propagation. The z position of the focusing lens is adjusted to maximize the detected signal, corresponding to the geometrical focus.

2. The focusing lens is scanned along the optical axis in 100 nm steps (corresponding to an effective movement of the focal zone of $100 \times n_0$ nm inside the material of linear refractive index n_0) over a total range of 200 μm around the geometrical focus z_g. At each position, a single xy image is acquired, resulting in a stack of 2,000 images. Examples of nonlinear propagation images for different z positions are shown in Supplementary Fig. 1(b). This image stack enables reconstruction of the on-axis fluence distribution.

3. The full procedure is repeated for various E_{in} values. The neutral density filters are adjusted to maintain a nearly constant maximum pixel value on the camera, and therefore optimal signal-to-noise ratio. As shown for GaAs and $\tau = 900$ fs in Supplementary Fig. 1(c) (pink points), the total filter transmission T_{tot} decreases linearly for low E_{in} (in the linear regime). In contrast, for high E_{in} (in the nonlinear regime), no more filters

need to be implemented in the microscope, and T_{tot} saturates to a specific value which depends on the tested material and laser conditions.

4. Standard image processing is performed, including noise removal and interpolation to replace outlier frames (e.g., due to laser timing jitter) using the average of the adjacent images.

5. To convert gray values to absolute fluence, the image stack acquired at the lowest E_{in} is assumed to correspond to linear Gaussian propagation. Under this assumption, the peak fluence at z_g is given by $F_{max} = 2T_F E_{in}/(T_{tot}\pi w_0^2)$, where $T_F = 1-(1-n_0)^2/(1+n_0)^2$ is the Fresnel transmission coefficient at normal incidence, and w_0 is the beam radius at $1/e^2$. All pixel values in the stack are scaled by F_{max}/F_{gv}, with F_{gv} being the peak gray value (in arbitrary units).

6. To reconstruct calibrated 3D fluence maps at higher E_{in}, we correct for changes in T_{tot} and in F_{gv} between each image stack. As shown in Supplementary Fig. 1(c) (yellow points), the trends in F_{max} and T_{tot} are inverted.

Once the 3D quantitative fluence distributions are obtained, the peak fluence F_p is finally defined as

$$F_p = \max_{E_{in}}(F_{max}) = \max_{x,y,z,E_{in}}(F). \qquad \text{(S1)}$$

Finally, normalized fluence distributions containing the optical axis [see Primary Manuscript, Figs. 2(b), 5(a), and 5(d)] are obtained by stacking fluence profiles—each divided by F_{max}—that intersect the center of the beam.

2.2 Necessary imaging conditions

Three conditions must be fulfilled for reliable nonlinear propagation imaging with high dynamic range. First, the numerical aperture of the imaging lens must be higher than the one of the focusing lens so that all angular components are imaged. This is guaranteed by the design of the optical arrangement for nonlinear propagation imaging, as we employed objective lenses with NA = 0.40 for focusing, and NA = 0.85 for imaging.

Second, for improved imaging performance, the camera response must be linear at the employed wavelength. To examine this, let us consider a laser pulse with a Gaussian temporal intensity profile $I(t) = I_0 \exp(-4\ln(2)t^2/\tau^2)$, where τ is the pulse duration defined at full width at half maximum (FWHM), $I_0 \propto E/(\pi w_0^2 \tau)$ is the maximum intensity, E is the pulse energy, and w_0 is the beam radius at $1/e^2$. The pixel amplitude A on the camera is proportional to the free-electron density n_e generated on the camera chip. In the general case of N-photon absorption, the following rate equation can be used to determine n_e

$$\frac{\partial n_e}{\partial t} = \frac{\beta_N I^N}{N\hbar\omega}, \qquad \text{(S2)}$$

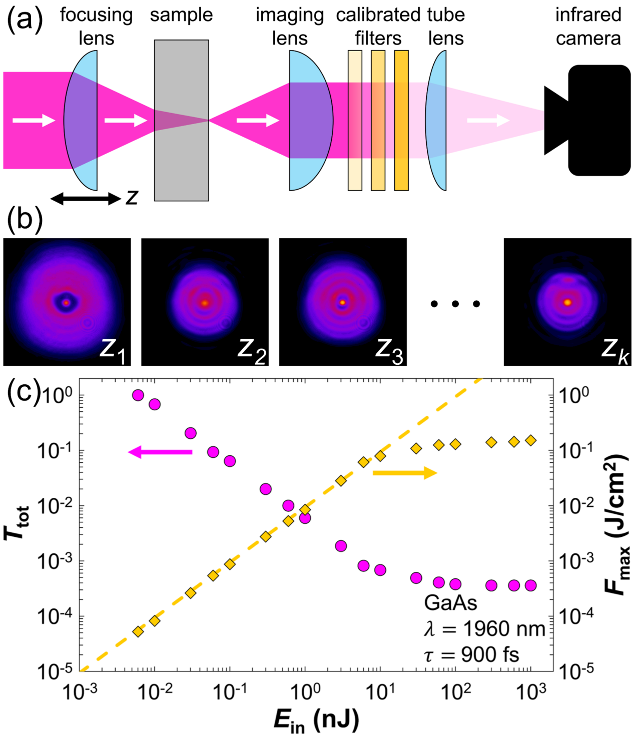

Supplementary Fig. 1 Nonlinear propagation imaging technique. (a) Schematic of the setup. (b) Examples of nonlinear propagation images recorded at different z positions of the focusing lens. (c) Evolution of the total filter transmission T_{tot}, and the maximum fluence F_{max} in GaAs (pulse duration $\tau = 900$ fs) as a function of the input pulse energy E_{in}. The dotted line corresponds to calculations of F_{max} in the linear regime (see details in Section Supplementary Note 3).

where β_N is the N-photon absorption coefficient, $\hbar$ is the reduced Planck constant, and ω is the angular frequency of the illuminating laser. The total free-electron density produced by the pulse thus reads

$$n_e \propto \left(\frac{E}{\pi w_0^2 \tau}\right)^N \int_{-\infty}^{+\infty} \exp\left(-4N\ln 2\frac{t^2}{\tau^2}\right) dt, \qquad \text{(S3)}$$

which leads to

$$n_e \propto \frac{E^N}{w_0^{2N}\tau^{N-1}}. \qquad \text{(S4)}$$

From Eq. (S4), one can note that the pixel amplitude A is independent of the pulse duration τ for linear absorption ($N = 1$), while it scales as $1/\tau$ for 2-photon absorption ($N = 2$). This provides a method to verify experimentally the linearity of the camera response. This method consists of measuring the evolution of the sum of the pixel amplitudes $\sum_{x,y} A(x, y)$ as a function of τ while all other parameters—in particular E and w_0—are kept constant. Experimental measurements at a wavelength of $\lambda = 1960$ nm are displayed in Supplementary Fig. 2 for the extended InGaAs array used in our study (in red), and for a Si-based camera (in blue). As expected, the signal detected on the Si camera decreases with τ. The deviation from the $1/\tau$ trend for the longest durations originates from the low signal-to-noise ratio. In contrast, the signal

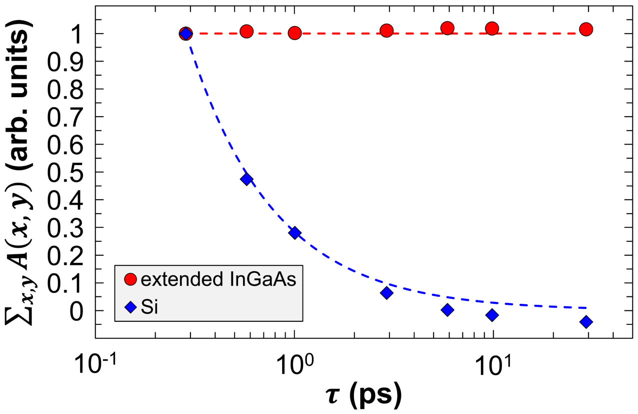

Supplementary Fig. 2 Evolution of the sum of the pixel amplitudes $\sum_{x,y} A(x,y)$ as a function of the pulse duration τ for an extended InGaAs (red) and a Si camera (blue). The points are measurements in the linear propagation regime at a wavelength of 1960 nm, and the curves correspond to trends expected from Eq. (S4). The pulse energy E and beam radius w_0 are kept constant in all measurements.

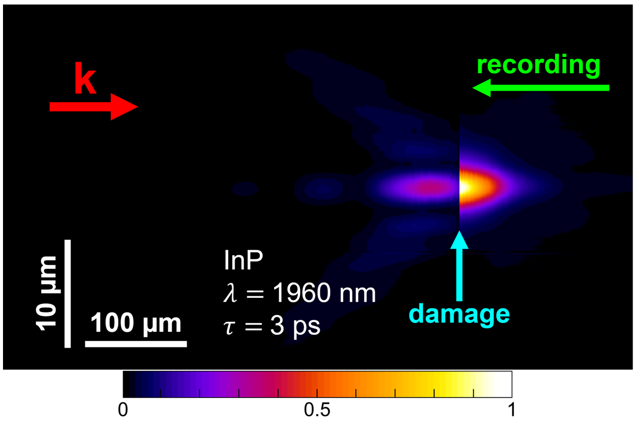

Supplementary Fig. 3 Normalized fluence distribution in the case of damage formed during nonlinear propagation imaging in InP for $E_{\text{in}} = 100$ nJ and $\tau = 3$ ps. The vector **k** indicates the direction of laser propagation. The green and blue arrows indicate the recording chronology and the plane where damage has been formed, respectively.

on the extended InGaAs array is independent of τ, thus showing that absorption at $\lambda = 1960$ nm on the camera chip is linear. While nonlinearly responding cameras ($N \geq 2$) can in principle be utilized for nonlinear propagation imaging to determine the maximum fluence [S82], this type of device underperforms linearly responding cameras ($N = 1$) for observing fine features in the 3D fluence distribution, which requires a high dynamic range.

The third condition to be fulfilled is that damage must not be induced in the sample during nonlinear propagation imaging. This has been systematically inspected after recording under white light illumination with the same microscope as for the measurements. When damage forms during the imaging procedure, it strongly affects the transmission by scattering and absorbing light. Moreover, during the recording of the subsequent images, the propagation can be strongly affected [S83]. The fluence distributions obtained are all the more complex to interpret when one keeps in mind that damage formed on the surface or in the bulk of the sample can grow on a pulse-to-pulse basis [S84, S85]. All these effects lead to the conclusion that damage must absolutely be avoided to get reliable experimental data, as illustrated in Supplementary Fig. 3 where damage formed during the imaging procedure drastically modifies light transmission for the subsequently recorded images.

2.3 Absence of cumulative effects

In our nonlinear propagation measurements, we implicitly assumed that the material excited by one pulse returns to rest before the subsequent pulse arrives, i.e., there are no cumulative effects. Let us examine the validity of this assumption. In semiconductors, the thermal diffusivity is two orders of magnitude higher than in dielectrics. Consequently, extreme repetition rates—far above the repetition rate $\Omega = 50$ kHz employed in our nonlinear propagation imaging measurements—are required to cause cumulative effects in these materials [S86]. To verify this, we have performed nonlinear propagation imaging for different repetition rates, adjusted with an acousto-optic modulator implemented in the laser. As shown in Supplementary Fig. 4, for $\Omega \leq 50$ kHz, the measured fluence distributions obtained in Si for high-intensity pulses ($E_{\text{in}} = 1000$ nJ and $\tau = 275$ fs) are independent of the repetition rate. We thus conclude that no cumulative effects take place during the nonlinear propagation measurements.

2.4 Repeatability

To estimate the repeatability of our experimental method, nonlinear propagation imaging has been carried out on two different days in identical conditions (Si, $\tau = 3$ ps). As shown in Supplementary Fig. 5, the measured F_{max} are similar over a broad range of input pulse energies E_{in}. The input pulse energy value for which the experimental data deviate from the linear propagation regime is the same for both data sets ($E_{\text{in}} = 30$ nJ). For $E_{\text{in}} \geq 100$ nJ where the saturation plateau for F_{max} is reached, the peak fluence is similar for both measurements ($F_p \approx 0.31$ J/cm^2), with a standard deviation of 15%. Therefore, F_p values are reported with an uncertainty of ±15%.

Supplementary Note 3 Linear propagation regime

3.1 Linear propagation calculations

Generally speaking, spatial beam distortions can be caused by spherical aberration due to refractive index mismatch at the air–material interface. Let us examine how pronounced the spherical aberration is for each material. To do so, linear propagation calculations have been carried out with the vectorial

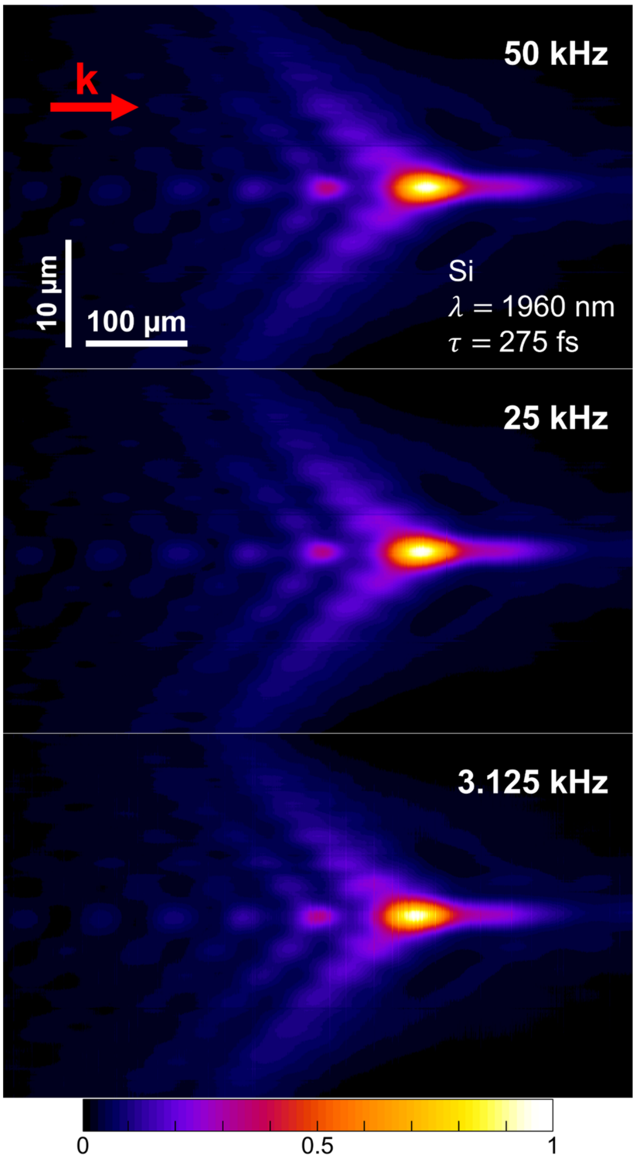

Supplementary Fig. 4 Normalized fluence distributions obtained at different repetition rates in Si for $E_{\rm in} = 1000$ nJ and $\tau = 275$ fs. The vector **k** indicates the direction of laser propagation. The spatial scales apply to all images.

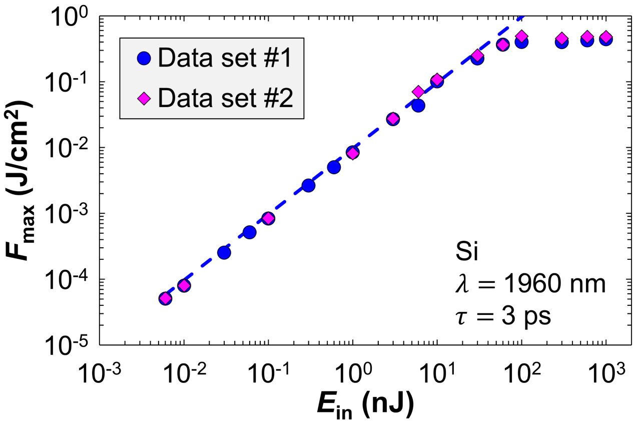

Supplementary Fig. 5 Evolution of the maximum fluence $F_{\rm max}$ measured in the bulk of Si on different days as a function of the input pulse energy $E_{\rm in}$. The pulse duration is $\tau = 3$ ps. The dashed blue line corresponds to the linear regime.

model *InFocus* [S87, S88]. The corresponding calculations of the spatial beam distribution along the optical axis z and the radial direction r are shown in Supplementary Fig. 6 (gray curves). As expected, higher refractive indices n_0 lead to a more extended focal zone along z, and have no influence along r. For each material, the experimental measurements for the lowest $E_{\rm in}$ values (red curves) are in excellent agreement with the calculations. The corresponding theoretical and experimental beam waist (w_0) and Rayleigh length (z_R) values are recapitulated in Supplementary Table 3. For both parameters, the calculations underestimate the experimental values by $< 3\%$. This minor difference could originate from experimental uncertainties, as well as the assumption that the focusing lens does not cause additional aberrations. From the $F(r)$ profiles displayed in Supplementary Fig. 6, one can distinguish side peaks at around ± 4 μm. The higher the amplitude of these peaks, the more pronounced the spherical aberration. Given that the amplitude of these peaks is $\approx 1\%$ of the main peak, we conclude that spherical aberration can be safely neglected, as additionally supported by the near symmetric $F(z)$ profiles.

Supplementary Table 3 Theoretical and experimental values for the beam radius at $1/e^2$ ($w_0^{\rm th}$ and $w_0^{\rm exp}$, respectively), and for the Rayleigh length ($z_R^{\rm th}$ and $z_R^{\rm exp}$, respectively).

Medium	$w_0^{\rm th}$ (μm)	$w_0^{\rm exp}$ (μm)	$z_R^{\rm th}$ (μm)	$z_R^{\rm exp}$ (μm)
Si	2.15	2.22	36.09	36.24
Ge	2.15	2.22	42.88	43.61
InP	2.15	2.20	32.79	33.09
GaAs	2.15	2.20	34.93	35.60

Increasing the numerical aperture NA of the focusing optics leads to higher energy deposition in semiconductors [S89]. However, even for aberration-corrected optical systems such as objective lenses, refraction at the air–solid interface with a high refractive index mismatch may cause severe spherical aberration. This aberration, which results in an asymmetric fluence distribution at the focus, is more pronounced for high refractive index mismatch, high NA, and a deep focus position inside the solid. To quantify the dependence of spherical aberration on NA, additional linear propagation calculations have been performed for different NAs with *InFocus*, with similar conditions as in the experiments ($\lambda = 1960$ nm, Gaussian beam with a diameter $\approx 60\%$ larger than the entrance pupil of the focusing optics, linear polarization, sample thickness of 500 μm). The considered material is Ge, as it shows the highest refractive index ($n_0 = 4.106$) among the semiconductors investigated. This implies that, if the spherical aberration is not pronounced in Ge, the same is true for Si, InP, and GaAs. To evaluate how pronounced the spherical aberration is, we define the

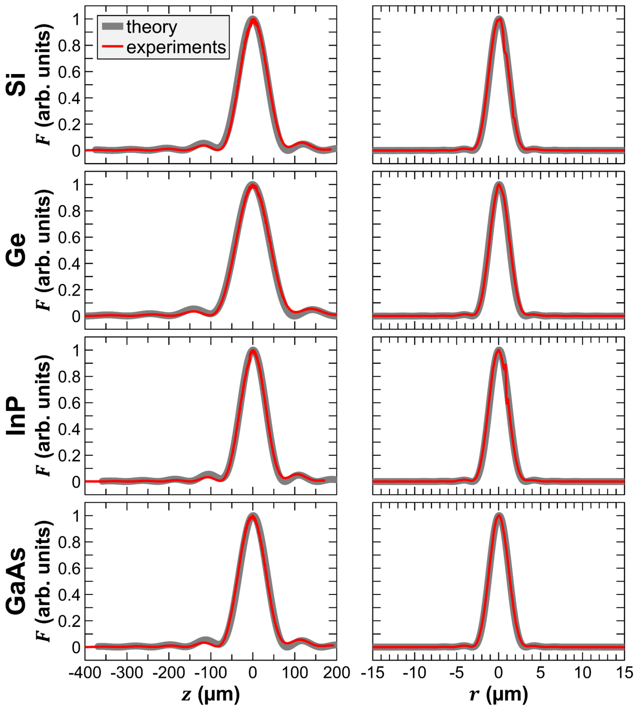

Supplementary Fig. 6 On-axis and radial fluence distributions in the linear regime for all semiconductors investigated. The gray curves are calculations with the vectorial model *InFocus* [S87, S88] for a focusing depth of 500 μm inside the material, and the red curves are experimental measurements.

asymmetricity parameter σ as

$$\sigma = \frac{1}{L}\left|\int_0^{z_g} F(z)dz - \int_{z_g}^{L} F(z)dz\right|, \quad \text{(S5)}$$

where F is the on-axis fluence, z_g is the on-axis position of the geometrical focus, and $L = 500$ μm is the sample thickness. By construction, $\sigma \approx 0$ when spherical aberration is negligible.

The evolution of σ as a function of NA is displayed in Supplementary Fig. 7. For NA ≤ 0.45, $\sigma \approx 0$, which demonstrates the absence of pronounced spherical aberration. In contrast, σ increases for NA ≥ 0.50, which indicates that spherical aberration can no longer be neglected. An important conclusion from the results in Supplementary Fig. 7 is that the objective lens of NA $= 0.40$ used in all experiments is an excellent compromise to obtain tight focusing without pronounced spherical aberration.

3.2 Maximum fluence in the linear regime

In the linear propagation regime, the maximum fluence $F_{\max}$ reached in the bulk of the considered medium can be simply expressed as a function of the input pulse energy E_{in}. Assuming a Gaussian fluence distribution

$$F(r) = F_{\max} e^{-\frac{2r^2}{w_0^2}}, \quad \text{(S6)}$$

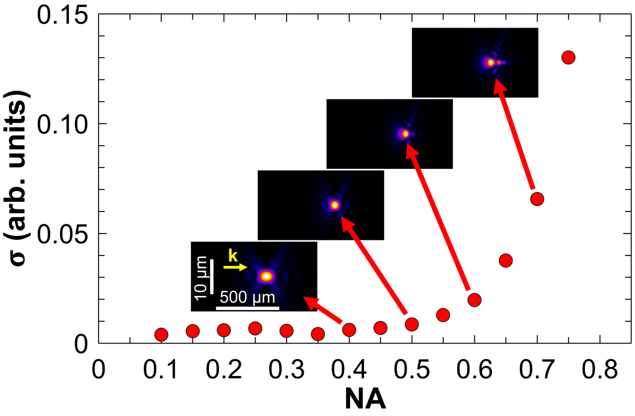

Supplementary Fig. 7 Evolution of the asymmetricity σ in Ge as a function of the numerical aperture NA. The insets show the result of calculations with the vectorial model *InFocus* [S87, S88] at different NAs. The vector **k** indicates the direction of laser propagation. The spatial scales apply to all images.

where w_0 is the beam radius at $1/e^2$, the input pulse energy E_{in} reads

$$\begin{aligned} T_F E_{\text{in}} &= \int_0^{2\pi}\int_0^{+\infty} F(r) r dr d\theta \\ &= 2\pi F_{\max}\int_0^{+\infty} r e^{-\frac{2r^2}{w_0^2}} dr \\ &= 2\pi F_{\max}\frac{w_0^2}{4}. \end{aligned} \quad \text{(S7)}$$

where $T_F = 1 - (1-n_0)^2/(1+n_0)^2$ is the Fresnel transmission coefficient at the air–medium interface at normal incidence. Thus, $F_{\max}$ reads

$$F_{\max} = \frac{2T_F E_{\text{in}}}{\pi w_0^2}. \quad \text{(S8)}$$

As shown in the Primary Manuscript, Fig. 2(d), calculations with Eq. (S8) are in excellent agreement with experimental measurements for low input pulse energy.

3.3 Group velocity dispersion

As shown in Supplementary Table 4, semiconductors are highly dispersive optical media. Let us examine if group velocity dispersion in these media can cause temporal pulse broadening. For bandwidth-limited Gaussian pulses, the pulse duration τ_{out} exiting the sample reads

$$\tau_{\text{out}} = \tau_{\text{in}}\sqrt{1 + \left(4\ln(2)\text{GVD}\frac{d}{\tau_{\text{in}}^2}\right)^2} \quad \text{(S9)}$$

where τ_{in} is the pulse duration entering the sample, GVD is the group velocity dispersion, and d is the sample thickness.

The bandwidth limit calculated as the Fourier transform of the experimentally measured spectrum is $\tau_{\text{in}} = 257$ fs. The difference between this theoretical limit and the minimum pulse duration of 275 fs determined

Supplementary Table 4 Group velocity dispersion GVD for all semiconductors investigated. The values are given for $\lambda \approx 1960$ nm.

Medium	GVD (fs^2/mm)	Reference
Si	830	[S90]
Ge	3474	[S91]
InP	1101	[S13]
GaAs	1053	[S18]

with autocorrelation originates from higher-order dispersion. Using the GVD values in Supplementary Table 4 and the sample thickness of $d = 500$ μm, the temporal pulse broadening $\Delta\tau = \tau_{\text{out}} - \tau_{\text{in}}$ according to Eq. (S9) is < 1 fs for all media. We thus conclude that group velocity dispersion can be safely neglected in all our experiments. This holds all the more for experiments at longer pulse durations. An important conclusion is that the differences observed in Si for up- and down-chirped 3-ps pulses [Primary Manuscript, Fig. 5(a)–(c)] cannot originate from group velocity dispersion.

3.4 Chirped temporal profiles

In the Primary Manuscript, Fig. 5(a)–(c), it was shown that the chirp has a significant effect on the peak fluence in the bulk of Si. To ensure that this effect is not caused by variations in the pulse temporal profile and asymmetry, we carried out fine spectral characterizations with a high-resolution optical spectrum analyzer (Yokogawa, AQ6375). To access the temporal profile, the typical spectrum shown in Supplementary Fig. 8(a) is converted into spectral amplitude in the frequency ν domain. Dispersion effects are accounted for by multiplying the spectral amplitude by $\exp(i\phi)$, where:

$$\phi = \sum_{k=0}^{+\infty} \frac{b_k}{k!} \left(\nu - \nu_0\right)^k . \qquad \text{(S10)}$$

Here, ν_0 denotes the carrier frequency. In practice, the dispersion coefficients b_k are set to 0, except for the group delay dispersion b_2. A pulse duration comparable to the Primary Manuscript, Fig. 5(a)–(c), is achieved for $b_2 = \pm\left(3 \times 10^{-12}\right)^2$ s^2 ($b_2 > 0$ and $b_2 < 0$ for up- and down-chirped pulses, respectively). Inverse Fourier transform is finally applied to the resulting complex spectral field in order to obtain the intensity in the time domain.

The calculated temporal profiles of 3-ps down- and up-chirped pulses are compared in Supplementary Fig. 8(b), where a bandwidth-limited pulse ($b_2 = 0$) is also displayed as a reference. As expected, both chirped pulses show mirrored temporal profiles. Moreover, it is important to note only minor asymmetry in these temporal profiles. This is a direct benefit of the smooth spectrum in Supplementary Fig. 8(a), showing very minor modulations. Therefore, the calculations in Supplementary Fig. 8(b) suggest that the increased peak fluence when using down-chirped pulses instead of up-chirped pulses [see Primary Manuscript, Fig. 5(a)–(c)] mainly originates from the different ionization dynamics which depend on the temporal sequence of spectral components.

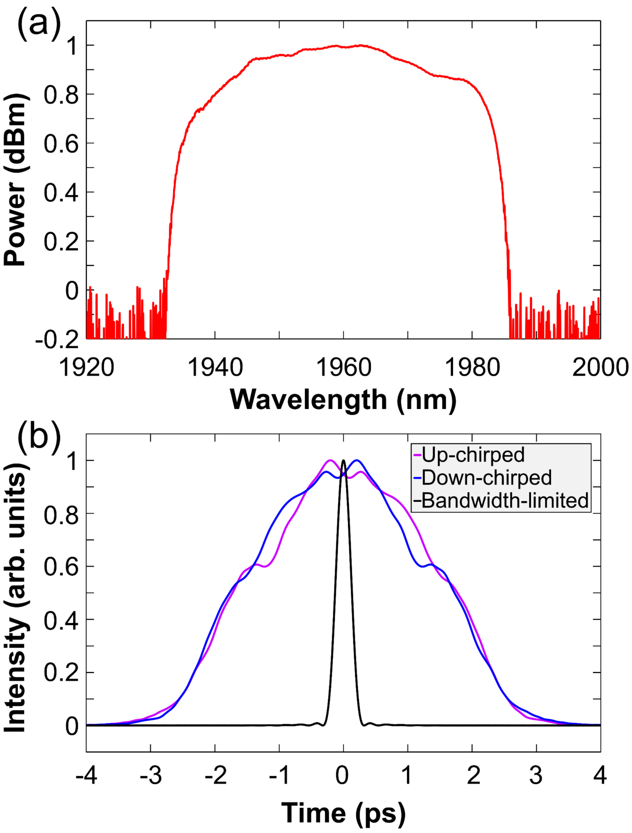

Supplementary Fig. 8 (a) Experimentally measured spectrum. (b) Calculated temporal profiles for a 3-ps down-chirped pulse [group delay dispersion $b_2 = -\left(3 \times 10^{-12}\right)^2$ s^2], a 3-ps up-chirped pulses [$b_2 = +\left(3 \times 10^{-12}\right)^2$ s^2], and a bandwidth-limited pulse ($b_2 = 0$).

Supplementary Note 4 Filamentation regime

4.1 Propagation morphology

As exemplified in the Primary Manuscript, Fig. 2(c), the morphology of the fluence distribution depends on the input pulse energy E_{in}. The morphologies obtained for different E_{in} and τ conditions have been examined for all semiconductors investigated (Supplementary Fig. 9). A common feature for all materials is that, for increased E_{in}, the propagation morphology changes consecutively from *grain of rice* to *egg* to *angel* to *pearl necklace*. Nevertheless, the transition E_{in} value between two morphologies strongly depends on the considered medium and pulse duration. This is in excellent agreement with the results shown in the Primary Manuscript, Fig. 3, where nonlinear refraction and absorption depend on τ.

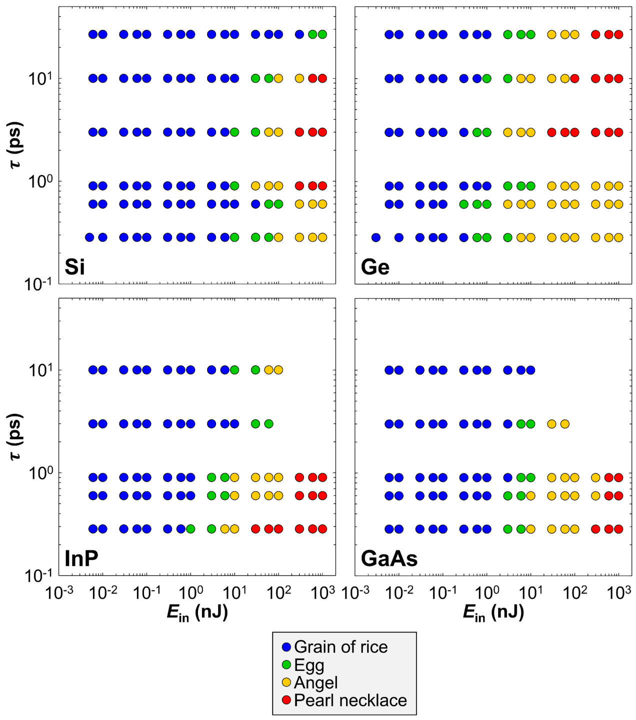

Supplementary Fig. 9 Morphology of the fluence distributions in all semiconductors investigated for different input pulse energy E_{in} and pulse duration τ.

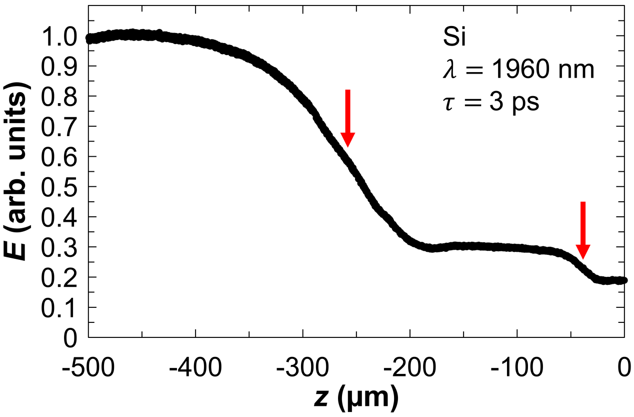

Supplementary Fig. 10 Evolution of the normalized energy E in Si for $E_{\text{in}} = 1\ \mu$J and $\tau = 3$ ps as a function of the on-axis distance z. The red arrows indicate the different inflection points for the same $E(z)$ profile.

4.2 Energy profiles

The evolution of the energy E as a function of the on-axis distance z [i.e., the $E(z)$ profile] is obtained by integrating the 3D fluence distributions $F(x, y, z)$ over x and y

$$E(z) = \iint_{\mathbb{R}^2} F(x, y, z)dxdy. \tag{S11}$$

As shown in the Primary Manuscript, Fig. 4(a) and (b), the $E(z)$ profiles are well-described by sigmoid functions defined as

$$E(z) = A - \frac{B}{1 + \exp\left(-\frac{z-D}{C}\right)}. \tag{S12}$$

The fitting parameters A and B define the energy before and after the interaction as $E(-\infty) = T_F E_{\text{in}} = A$ and $E(+\infty) = A - B$, respectively. The fraction of absorbed energy is defined as $f_E = 1 - E(+\infty)/E(-\infty) = B/A$. The steepness of the sigmoid is inversely proportional to the parameter C, which can be defined as the characteristic absorption length ($C = L_{\text{abs}}$). Finally the parameter D defines the position of the inflection point. A necessary fitting condition is that the $E(z)$ profile must exhibit a single inflection point. A counter-example for high E_{in} is shown in Supplementary Fig. 10, where two inflection points exist, invalidating the sigmoid fit defined in Eq. (S12). This situation of multiple inflection points on the $E(z)$ profile is generally obtained at high energy, where the filament exhibits an *angel* or *pearl necklace* morphology (see Section 4.1).

As shown in the Primary Manuscript, Fig. 4(c) and (d), f_E and L_{abs} both scale logarithmically with E_{in}, and also with $1/\tau$. This has been examined by fitting the experimental data as

$$\begin{cases} f_E = a_{f_E} \ln(\tau) + b_{f_E} \ln(E_{\text{in}}) + c_{f_E}, \\ L_{\text{abs}} = a_{L_{\text{abs}}} \ln(\tau) + b_{L_{\text{abs}}} \ln(E_{\text{in}}) + c_{L_{\text{abs}}}. \end{cases} \tag{S13}$$

The corresponding fitting coefficients are given in Supplementary Table 5. The striking feature highlighted by the nearly parallel planes in the Primary Manuscript, Fig. 4(c) is that the coefficients a_{f_E} and b_{f_E} are very similar for all materials. The material-dependent coefficient c_{f_E} represents the threshold for energy absorption. In contrast, all fitting coefficients for L_{abs} strongly differ from one material to another. The evolution of f_E and L_{abs} as a function of E_{in} and the corresponding fits according to Eq. (S13) are displayed as two-dimensional graphs in Figs. 11 and 12, respectively.

Supplementary Table 5 Fitting coefficients a_{f_E}, b_{f_E}, c_{f_E}, $a_{L_{\text{abs}}}$, $b_{L_{\text{abs}}}$, and $c_{L_{\text{abs}}}$ in Eq. (S13) for all tested semiconductors.

Medium	Si	Ge	InP	GaAs
a_{f_E}	-0.11 ± 0.01	-0.15 ± 0.01	-0.14 ± 0.01	-0.12 ± 0.02
b_{f_E}	0.16 ± 0.01	0.16 ± 0.01	0.15 ± 0.01	0.17 ± 0.01
c_{f_E}	-0.17 ± 0.03	0.57 ± 0.02	-0.04 ± 0.03	-0.05 ± 0.04
$a_{L_{\text{abs}}}$	-3.13 ± 0.88	-8.09 ± 1.70	-4.42 ± 1.13	-6.72 ± 1.87
$b_{L_{\text{abs}}}$	3.30 ± 1.18	8.56 ± 1.32	4.65 ± 1.06	7.82 ± 1.27
$c_{L_{\text{abs}}}$	1.73 ± 4.65	35.23 ± 2.12	-0.94 ± 3.45	-7.67 ± 4.55

4.3 Critical power for nonlinearities

Experimental determination

The on-axis distributions calculated in Supplementary Fig. 6 serve as a benchmark for determining the input pulse energy E_{cr}, which delimits the linear and the nonlinear propagation regimes. As exemplified in Supplementary Fig. 13(a) for $\tau = 900$ fs, the on-axis fluence profiles are determined for various E_{in} values,

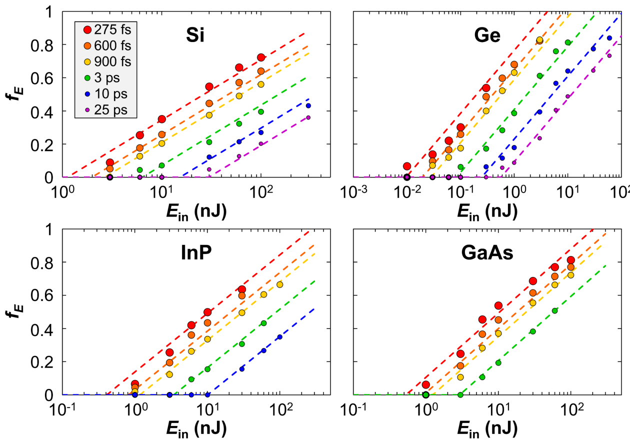

Supplementary Fig. 11 Evolution of the fraction of absorbed energy f_E as a function of $E_{\rm in}$ for different pulse durations τ and semiconductors. The dashed lines correspond to calculations according to Eq. (S13) with the a_{f_E}, b_{f_E}, and c_{f_E} coefficients given in Supplementary Table 5.

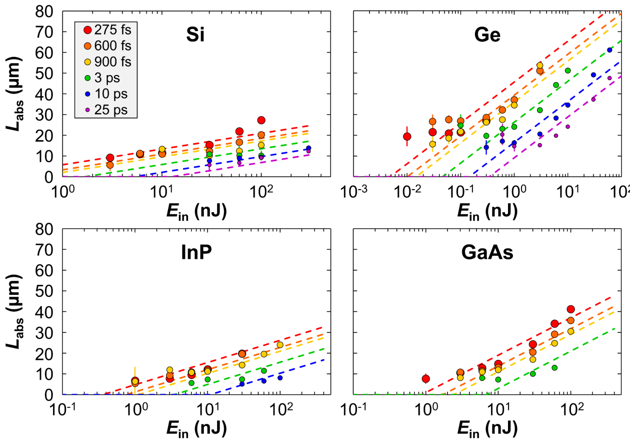

Supplementary Fig. 12 Evolution of the characteristic absorption length $L_{\rm abs}$ as a function of $E_{\rm in}$ for different pulse durations τ and semiconductors. The dashed lines correspond to calculations according to Eq. (S13) with the $a_{L_{\rm abs}}$, $b_{L_{\rm abs}}$, and $c_{L_{\rm abs}}$ coefficients given in Supplementary Table 5.

and directly compared to calculations performed with *InFocus* (see Section Supplementary Note 3). For pulse energies $E_{\rm in} \leq 3$ nJ, the measurements are in excellent agreement with calculations. However, for higher $E_{\rm in}$ values, the experimental profiles deviate from the linear regime due to Kerr and plasma effects. We introduce the difference Δ between the experimental and theoretical profiles as

$$\Delta = \int_{-\infty}^{+\infty} \left| F^{\rm exp}(z) - F^{\rm th}(z) \right| dz, \qquad \text{(S14)}$$

where $F^{\rm exp}$ and $F^{\rm th}$ are the experimental and theoretical on-axis fluence profiles, respectively. The model *InFocus* is used to calculate $F^{\rm th}$ with 250-nm steps along the z-axis.

An example of the evolution of Δ as a function of $E_{\rm in}$ is displayed in Supplementary Fig. 13(b). For $E_{\rm in} \leq 3$ nJ, Δ is nearly constant, again highlighting that the propagation regime is linear. In contrast, Δ increases with $E_{\rm in} > 3$ nJ, which indicates that the propagation regime is nonlinear. The critical pulse energy $E_{\rm cr}$, which delimits the two propagation regimes is thus determined as the average between the highest input pulse energy for which the propagation is linear [$E_{\rm in}^- = 3$ nJ in Supplementary Fig. 13(b)], and the lowest input pulse energy for which the propagation is nonlinear [$E_{\rm in}^+ = 6$ nJ in Supplementary Fig. 13(b)]. The effective critical power shown in the Primary Manuscript, Fig. 3(b) is evaluated for different pulse durations τ as

$$P_{\rm cr}^{\rm eff} = 0.88 \frac{T_F E_{\rm cr}}{\tau}, \qquad \text{(S15)}$$

where T_F is the Fresnel transmission coefficient. The uncertainty in $P_{\rm cr}^{\rm eff}$ values is calculated as

$$\varepsilon = 0.88 \frac{T_F |E_{\rm in}^+ - E_{\rm cr}|}{\tau} = 0.88 \frac{T_F |E_{\rm in}^- - E_{\rm cr}|}{\tau}. \qquad \text{(S16)}$$

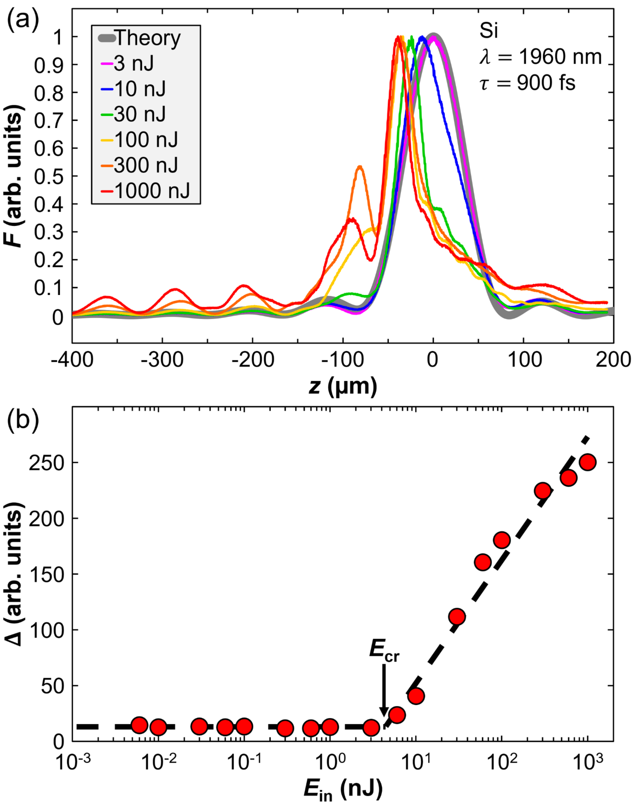

Supplementary Fig. 13 Determination of the critical pulse energy $E_{\rm cr}$ which delimits the linear and the nonlinear propagation regimes. (a) On-axis normalized fluence distributions in Si for different input pulse energies $E_{\rm in}$. The gray curve has been calculated in the linear propagation regime with the vectorial model *InFocus* [S87, S88], and the other curves have been obtained experimentally for $\tau = 900$ fs. (b) Evolution of Δ according to Eq. (S14) between theory and experiments as a function of the input pulse energy $E_{\rm in}$. The dashed lines are guides for the eye.

Temporal scaling laws

In the Primary Manuscript, we have shown that the measured critical power $P_{\rm cr}^{\rm eff}$ decreases for increasing pulse duration τ [see Fig. 3(b)]. To give a physical interpretation of this temporal dependence, let us consider the nonlinear polarization $P_{\rm nl}$. Considering only the Kerr effect, and neglecting third harmonic generation, $P_{\rm nl}$ can be written as

$$P_{\rm nl}(t) = 2n_0\varepsilon_0 n_2^{\rm eff}(t)I(t)E(t), \tag{S17}$$

where ε_0 is the vacuum permittivity, n_0 is the linear refractive index, $I(t)$ is the intensity, $E(t)$ is the magnitude of the electric field, and $n_2^{\rm eff}(t)$ is the time-dependent effective nonlinear index. The temporal dependence of $n_2^{\rm eff}(t)$ implies a delayed medium response, which can be written in the most generic form as

$$n_2^{\rm eff}(t) = n_2\frac{\int_{-\infty}^{t} H(t-t')I(t')dt'}{I(t)} = n_2\frac{H\otimes I}{I(t)}, \tag{S18}$$

where $H(t)$ is the medium response function which must satisfy the causality condition $H(t<0)=0$, and be normalized to unity so that $\int_0^\infty H(t)dt = 1$. In Eq. (S18), n_2 is a nonlinear index corresponding to an infinitely long laser pulse compared to the characteristic medium response time τ_r. Indeed, for $\tau \gg \tau_r$, $H(t)$ can be considered as a Dirac delta function, resulting in $H\otimes I = I$, which leads to $n_2^{\rm eff} = n_2$.

Let us first consider a Gaussian medium response

$$H(t) = \frac{2\eta}{\sqrt{\pi}\tau_r}\exp\left(-\eta^2\frac{t^2}{\tau_r^2}\right), \tag{S19}$$

where τ_r is the characteristic response time of the medium, and η is a shape factor. Below, we set $\eta = 2\sqrt{\ln 2}$, so that τ_r is defined at FWHM. Furthermore, let us assume a Gaussian intensity profile $I(t)$

$$I(t) = I_0\exp\left(-\eta^2\frac{t^2}{\tau^2}\right), \tag{S20}$$

where I_0 is the peak intensity, and τ is the duration at FWHM. Using Eqs. (S19) and (S20), the convolution in Eq. (S18) can be expressed analytically as

$$H\otimes I = \frac{I_0}{\sqrt{1+\frac{\tau_r^2}{\tau^2}}}\exp\left(-\eta^2\frac{t^2}{\tau_r^2+\tau^2}\right)$$
$$\operatorname{erfc}\left(-\eta\frac{\tau_r}{\tau}\frac{t}{\sqrt{\tau_r^2+\tau^2}}\right), \tag{S21}$$

where erfc $= 1 -$ erf is the complementary error function. From Eqs. (S17), (S18) and (S20), one can qualitatively conclude that the propagation is mainly influenced by the effective nonlinear refractive index $n_2^{\rm eff}$ at $t=0$ for which the intensity reaches its maximum value I_0—and thus, the nonlinear polarization $P_{\rm nl}$ is the strongest. Combining Eqs. (S18) and (S21) at $t=0$, the effective nonlinear index reads

$$n_2^{\rm eff}(t=0) = \frac{n_2}{\sqrt{1+\frac{\tau_r^2}{\tau^2}}}, \tag{S22}$$

which leads to the following simple expression for the effective critical power $P_{\rm cr}^{\rm eff}$ as the function of τ

$$P_{\rm cr}^{\rm eff}(\tau) = P_{\rm cr}\sqrt{1+\frac{\tau_r^2}{\tau^2}}, \tag{S23}$$

where $P_{\rm cr} = \alpha\lambda^2/(4\pi n_0 n_2)$ is the critical power obtained for long pulses for which the effect of the delayed medium response is negligible, and $\alpha = 1.8962$.

The temporal evolution of the normalized convolution $H\otimes I/I_0$ according to Eq. (S21) is shown in Supplementary Fig. 14 for the experimental pulse durations τ investigated. The medium response is assumed to be Gaussian, with a characteristic time $\tau_r = 4.2$ ps corresponding to the average value for all semiconductors investigated (see below). The value of $H\otimes I/I_0$ at $t=0$—and thus, the effective nonlinear refractive index $n_2^{\rm eff}$—increases with the pulse duration. As $P_{\rm cr}^{\rm eff} \propto 1/n_2^{\rm eff}$, one can conclude that the effective critical power decreases with the pulse duration, in good agreement with the experimental results in the Primary Manuscript, Fig. 3(b). While quantitative differences may arise when selecting different shapes for the response function $H(t)$, it does not qualitatively impact on the trend that $P_{\rm cr}^{\rm eff}$ decreases with τ.

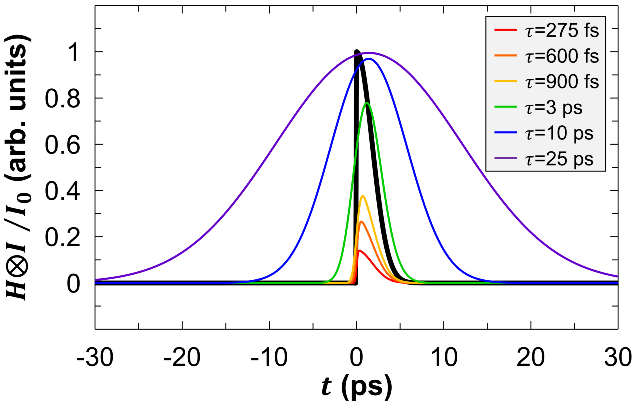

Supplementary Fig. 14 Time dependence of the normalized convolution integral $H\otimes I/I_0$ according to Eq. (S21) for the experimental pulse durations τ. The black curve is the normalized Gaussian response function $H(t)$ [Eq. (S19)], with a response time $\tau_r = 4.2$ ps.

For the same Gaussian intensity profile $I(t)$ defined in Eq. (S20), one could also consider an exponentially decaying response function

$$H(t) = \frac{\eta}{\tau_r}\exp\left(-\eta\frac{t}{\tau_r}\right), \tag{S24}$$

In this case the convolution in Eq. (S18) becomes

$$H\otimes I = I_0\frac{\sqrt{\pi}}{2}\frac{\tau}{\tau_r}\exp\left(\frac{1}{4}\frac{\tau^2}{\tau_r^2} - \eta\frac{t}{\tau_r}\right)$$
$$\operatorname{erfc}\left(\frac{1}{2}\frac{\tau}{\tau_r} - \frac{\eta^2}{2}\frac{t}{\tau}\right), \tag{S25}$$

and the corresponding effective nonlinear index n_2^{eff} at $t=0$ becomes

$$n_2^{\text{eff}}(t=0) = n_2 \frac{\sqrt{\pi}}{2}\frac{\tau}{\tau_r}\exp\left(\frac{1}{4}\frac{\tau^2}{\tau_r^2}\right)\text{erfc}\left(\frac{1}{2}\frac{\tau}{\tau_r}\right). \tag{S26}$$

Therefore, the effective critical power $P_{\text{cr}}^{\text{eff}}$ takes the following form

$$P_{\text{cr}}^{\text{eff}}(\tau) = P_{\text{cr}}\frac{2}{\sqrt{\pi}}\frac{\tau_r}{\tau}\frac{\exp\left(-\frac{1}{4}\frac{\tau^2}{\tau_r^2}\right)}{\text{erfc}\left(\frac{1}{2}\frac{\tau}{\tau_r}\right)}. \tag{S27}$$

To estimate the response time τ_r for different semiconductors, nonlinear curve fitting of the experimental data presented in the Primary Manuscript, Fig. 3(b) is applied for a Gaussian response using Eq. (S23). The chosen values for P_{cr} correspond to the effective critical powers determined with the longest pulses with which measurements could be performed. The obtained fits are superimposed with the experimental $P_{\text{cr}}^{\text{eff}}$ values in the Primary Manuscript, Fig. 3(b). For all semiconductors, the trends given by Eq. (S23) are well reproduced, allowing us to conclude that the major physical mechanisms responsible for the decrease of $P_{\text{cr}}^{\text{eff}}$ with increasing pulse duration τ are correctly described. Similar results (not shown here) have been obtained using Eq. (S27) assuming an exponentially decaying medium response. As shown in Supplementary Table 6, for both considered response shapes, the response times τ_r for all materials are on the same order of magnitude.

Supplementary Table 6 Medium response time τ_r (in picoseconds) obtained by nonlinear curve fitting of the experimental $P_{\text{cr}}^{\text{eff}}$ data for Gaussian [Eq. (S19)], and exponentially decaying [Eq. (S24)] response function $H(t)$.

$H(t)$	Si	Ge	InP	GaAs
Gaussian	2.94	11.41	1.72	0.67
exponential decay	2.22	9.19	1.28	0.47

4.4 Multi-photon absorption coefficient

To determine the effective N-photon absorption coefficients β_N^{eff}, we apply our recently developed approach relying on a modified Marburger formula, where power losses are accounted for [S92]. The nonlinear focal shift Δz reads

$$\Delta z = -\left(d - \frac{1}{1/d + 1/z_{\text{nl}}}\right), \tag{S28}$$

where $d = 500$ μm is the sample thickness, and z_{nl} is given by Marburger formula [S93]

$$z_{\text{nl}} = \frac{0.367 k_0 a_0^2}{\sqrt{\left(\sqrt{P/P_{\text{cr}}} - 0.852\right)^2 - 0.0219}}, \tag{S29}$$

where $k_0 = n_0\omega_0/c$ is the wave number with n_0, ω_0 and c corresponding to the linear refractive index, the pulse central angular frequency, and the speed of light in vacuum, respectively, $a_0 = d\tan(\arcsin(\text{NA}/n_0))$ is the beam radius at the entrance of the sample, NA $= 0.40$ is the numerical aperture of the focusing lens, and P is the peak power. Here, P_{cr} is taken as the effective critical power determined with the method described in Section 4.3.

To evaluate the peak power P at the exit surface after propagation losses, we use the time-independent nonlinear Schrödinger equation

$$\frac{\partial E}{\partial z} = \frac{i}{2k_0}\Delta_\perp E - \frac{\beta_N^{\text{eff}}}{4} I^{N-1} E, \tag{S30}$$

where E is the magnitude of the electric field, and I is the intensity. The first and second terms on the right-hand side of Eq. (S30) correspond to diffraction and N-photon absorption, respectively. Given the tight focusing conditions that we use, the dominant propagation mechanism is diffraction, and multi-photon absorption mainly leads to a decrease of the peak intensity without altering the propagation. The assumption that diffraction and multi-photon absorption act independently should hold true as long as the peak power P does not exceed the critical power P_{cr} by several orders of magnitude. In this regime, one can safely assume that the pulse duration τ is constant. Under these assumptions, one can define the electric fields E_d and E_a (the subscripts d and a standing for diffraction and absorption, respectively), which satisfy

$$\frac{\partial E_d}{\partial z} = \frac{i}{2k_0}\Delta_\perp E_d, \tag{S31}$$

and

$$\frac{\partial E_a}{\partial z} = -\frac{\beta_N^{\text{eff}}}{4} I^{N-1} E_a. \tag{S32}$$

Multiplying Eqs. (S31) and (S32) by the complex conjugates E_d^* and E_a^*, respectively, the solutions I_d and I_a for the differential equations read

$$I_d(x,y,z) = \frac{a_0^2}{a(z)^2}\exp\left(-\frac{x^2+y^2}{a(z)^2}\right), \tag{S33}$$

and

$$I_a(z) = \frac{I_0}{\left[1 + \frac{N-1}{2}\beta_N^{\text{eff}} I_0^{N-1} z\right]^{\frac{1}{N-1}}}, \tag{S34}$$

where $a(z)$ is the beam radius at the on-axis position z.

Finally, the peak power $P(z)$ can be expressed as a function of the intensity $I = I_d I_a$ as

$$\begin{aligned} P(z) &= \iint_{\mathbb{R}^2} I(x,y,z)\,dxdy \\ &= \frac{a_0^2}{a(z)^2} I_a(z) \iint_{\mathbb{R}^2} \exp\left(-\frac{x^2+y^2}{a(z)^2}\right) dxdy \\ &= \pi a_0^2 I_a(z) \\ &= \frac{P_0}{\left[1 + \frac{N-1}{2}\beta_N^{\text{eff}}\left(\frac{P_0}{\pi a_0^2}\right)^{N-1} z\right]^{\frac{1}{N-1}}}, \end{aligned} \tag{S35}$$

where $P_0 = I_0 \pi a_0^2$ is the power right after the entrance surface of the material considered. To extract β_N^{eff}, nonlinear curve fitting of experimental Δz data is applied using Eqs. (S28), (S29) where $P_{\text{cr}}^{\text{eff}}$ is determined following the method detailed in Section 4.3, and (S35).

The experimental values Δz obtained in Ge and InP are compared in Supplementary Fig. 15 to calculations using the Marburger formula where power losses are [Eqs. (S28), (S29) and (S35)] and are not [Eqs. (S28) and (S29) for $P = P_0$] accounted for. Here, $\Delta z = z_{\text{max}} - z_g$, where z_{max} corresponds to the position for which the maximum fluence F_{max} is reached, and z_g corresponds to the geometrical focus. When power losses are not taken into account, the traditional Marburger formula can be used to estimate Δz for about one order of magnitude above the critical power. However, this simple approach catastrophically fails to reproduce the experimental trends for higher power values. In contrast, our approach where power losses are accounted for allows us to fit the experimental data over several orders of magnitude. It is worth noting the applicability of our method for different multi-photon absorption orders (2PA and 3PA for Ge and InP, respectively). This approach allows us to determine β_2^{eff} and β_3^{eff} shown in the Primary Manuscript, Fig. 3(c). Accounting for the uncertainty in $P_{\text{cr}}^{\text{eff}}$, the error on β_2^{eff} and β_3^{eff} values is estimated at ±50%.

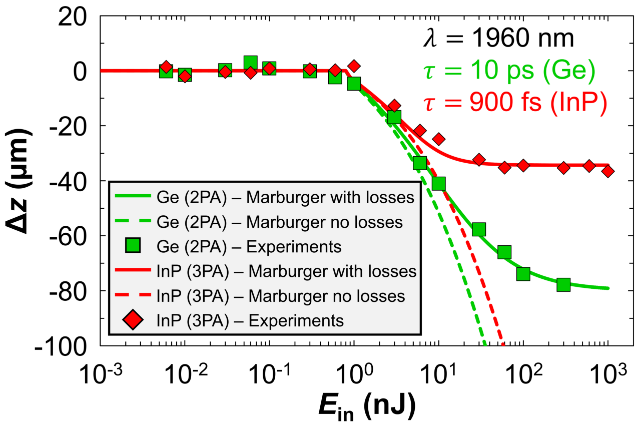

Supplementary Fig. 15 Evolution of the nonlinear focal shift Δz as a function of the input pulse energy E_{in} for Ge (green, 2PA, $\tau = 10$ ps) and InP (red, 3PA, $\tau = 900$ fs). The solid and dashed curves are calculations according to Marburger formulas accounting for and neglecting nonlinear power losses, respectively.

To apply the method we propose for determining the effective multi-photon absorption coefficient, a necessary condition is $\Delta z < 0$, i.e., the focus shifts upstream the laser with respect to the geometrical focus. Moreover, $|\Delta z|$ must monotonically increase with E_{in}. While these conditions are satisfied for the wide majority of our measurements, we noticed that these conditions are not fulfilled for Ge when employing sub-picosecond pulses. As shown in Supplementary Fig. 16, the two conditions are fulfilled for pulse durations $\tau \geq 3$ ps. However, for $\tau = 900$ fs, positive Δz values are measured. This suggests that, in this regime, the assumption that multi-photon absorption does not affect propagation [Eqs. (S31) and (S32)] is not valid anymore, and plasma defocusing may play an important role even for powers right above $P_{\text{cr}}^{\text{eff}}$. This is all the more confirmed when the pulse duration is further decreased to $\tau = 600$ and 275 fs. In these high input intensity conditions, Δz first decreases with E_{in}, and then increases when E_{in} is further increased. This highlights the complexity of ultrafast laser-Ge interaction. As a direct consequence, β_2^{eff} cannot be extracted from data obtained in these conditions.

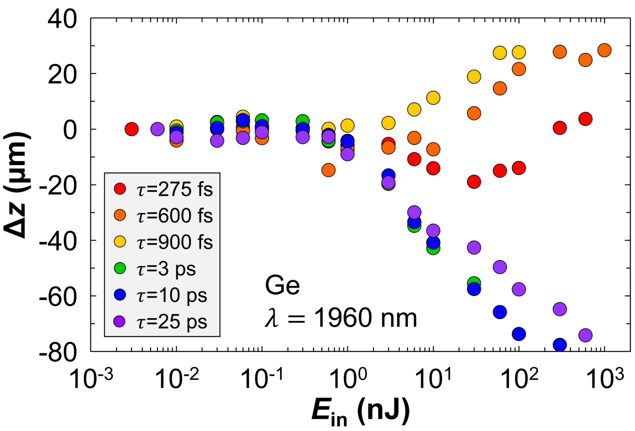

Supplementary Fig. 16 Evolution of the nonlinear focal shift Δz in Ge as a function of the input pulse energy E_{in} for various pulse durations τ.

4.5 Summary tables

The main results on key nonlinear parameters obtained for various laser conditions in the Primary Manuscript are recapitulated in Supplementary Tables 7, 8, 9, and 10, for Si, Ge, InP, and GaAs, respectively.

Supplementary Table 7 Results obtained in Si. The values of the peak fluence F_p, the effective critical power P_{cr}^{eff}, and the multi-photon absorption coefficient β_N^{eff} are shown for different pulse durations τ, and chirps.

λ (nm)	N	τ (ps)	chirp	F_p (J/cm^2)	P_{cr}^{eff} (kW)	β_N^{eff} (m^{2N-3}/W^{N-1})
1960	2	0.275	unchirped	$(3.9 \pm 0.58) \times 10^{-1}$	$(10 \pm 3.3) \times 10^{0}$	$(5.7 \pm 2.8) \times 10^{-10}$
1960	2	0.6	up	$(1.3 \pm 0.20) \times 10^{0}$	$(8.2 \pm 2.0) \times 10^{0}$	$(2.5 \pm 1.2) \times 10^{-9}$
1960	2	0.9	up	$(5.8 \pm 0.87) \times 10^{-1}$	$(3.1 \pm 1.0) \times 10^{0}$	$(1.2 \pm 0.61) \times 10^{-9}$
1960	2	3	up	$(4.9 \pm 0.74) \times 10^{-1}$	$(1.6 \pm 0.41) \times 10^{0}$	$(1.8 \pm 0.92) \times 10^{-9}$
1960	2	3	down	$(1.2 \pm 0.18) \times 10^{0}$	$(1.6 \pm 0.41) \times 10^{0}$	$(3.0 \pm 1.5) \times 10^{-9}$
1960	2	10	up	$(2.0 \pm 0.31) \times 10^{0}$	$(0.5 \pm 0.12) \times 10^{0}$	$(6.0 \pm 3.0) \times 10^{-9}$
1960	2	25	up	$(3.4 \pm 0.52) \times 10^{0}$	$(1.1 \pm 0.4) \times 10^{0}$	$(2.7 \pm 1.4) \times 10^{-8}$

Supplementary Table 8 Results obtained in Ge. The values of the peak fluence F_p, the effective critical power P_{cr}^{eff}, and the multi-photon absorption coefficient β_N^{eff} are shown for different pulse durations τ, and chirps.

λ (nm)	N	τ (ps)	chirp	F_p (J/cm^2)	P_{cr}^{eff} (kW)	β_N^{eff} (m^{2N-3}/W^{N-1})
1960	2	0.275	unchirped	$(1.2 \pm 0.18) \times 10^{-2}$	$(4.0 \pm 2.0) \times 10^{1}$	–
1960	2	0.6	up	$(1.9 \pm 0.29) \times 10^{-2}$	$(1.8 \pm 0.92) \times 10^{-1}$	–
1960	2	0.9	up	$(5.0 \pm 0.75) \times 10^{-2}$	$(4.9 \pm 1.2) \times 10^{-1}$	–
1960	2	3	up	$(2.1 \pm 0.31) \times 10^{-2}$	$(8.3 \pm 2.8) \times 10^{-2}$	$(2.1 \pm 1.0) \times 10^{-8}$
1960	2	10	up	$(1.9 \pm 0.29) \times 10^{-2}$	$(4.4 \pm 1.1) \times 10^{-2}$	$(2.4 \pm 1.2) \times 10^{-8}$
1960	2	25	up	$(5.5 \pm 0.83) \times 10^{-2}$	$(1.8 \pm 0.44) \times 10^{-2}$	$(1.0 \pm 0.51) \times 10^{-7}$

Supplementary Table 9 Results obtained in InP. The values of the peak fluence F_p, the effective critical power P_{cr}^{eff}, and the multi-photon absorption coefficient β_N^{eff} are shown for different wavelengths λ, multi-photon absorption orders N, pulse durations τ, and chirps.

λ (nm)	N	τ (ps)	chirp	F_p (J/cm^2)	P_{cr}^{eff} (kW)	β_N^{eff} (m^{2N-3}/W^{N-1})
1960	3	0.275	unchirped	$(1.1 \pm 0.17) \times 10^{-1}$	$(1.9 \pm 0.47) \times 10^{0}$	$(1.9 \pm 0.96) \times 10^{-21}$
1960	3	0.6	up	$(1.1 \pm 0.17) \times 10^{-1}$	$(8.6 \pm 2.2) \times 10^{-1}$	$(5.4 \pm 2.7) \times 10^{-21}$
1960	3	0.9	up	$(1.5 \pm 0.22) \times 10^{-1}$	$(5.7 \pm 1.4) \times 10^{-1}$	$(1.1 \pm 0.54) \times 10^{-20}$
1555	2	0.9	unchirped	$(1.0 \pm 0.15) \times 10^{-2}$	$(5.7 \pm 1.4) \times 10^{-2}$	$(7.8 \pm 3.9) \times 10^{-8}$
1960	3	3	up	$(5.1 \pm 0.76) \times 10^{-1}$	$(4.3 \pm 2.2) \times 10^{-1}$	$(1.6 \pm 0.81) \times 10^{-19}$
1960	3	10	up	$(6.5 \pm 0.98) \times 10^{-1}$	$(2.9 \pm 0.97) \times 10^{-1}$	$(7.6 \pm 3.8) \times 10^{-20}$

Supplementary Table 10 Results obtained in GaAs. The values of the peak fluence F_p, the effective critical power P_{cr}^{eff}, and the multi-photon absorption coefficient β_N^{eff} are shown for different wavelengths λ, multi-photon absorption orders N, pulse durations τ, and chirps.

λ (nm)	N	τ (ps)	chirp	F_p (J/cm^2)	P_{cr}^{eff} (kW)	β_N^{eff} (m^{2N-3}/W^{N-1})
1960	3	0.275	none	$(9.3 \pm 1.4) \times 10^{-2}$	$(1.8 \pm 0.45) \times 10^{0}$	$(8.5 \pm 4.3) \times 10^{-22}$
1960	3	0.6	up	$(8.7 \pm 1.3) \times 10^{-2}$	$(2.1 \pm 1.0) \times 10^{0}$	$(5.0 \pm 2.5) \times 10^{-22}$
1960	3	0.9	up	$(1.5 \pm 0.23) \times 10^{-1}$	$(1.4 \pm 0.69) \times 10^{0}$	$(1.5 \pm 0.77) \times 10^{-21}$
1555	2	0.9	unchirped	$(8.5 \pm 1.3) \times 10^{-3}$	$(1.4 \pm 0.69) \times 10^{-1}$	$(7.5 \pm 3.8) \times 10^{-8}$
1960	3	3	up	$(3.7 \pm 0.55) \times 10^{-1}$	$(9.4 \pm 3.1) \times 10^{-1}$	$(2.9 \pm 1.5) \times 10^{-21}$

4.6 Applications beyond laser direct writing

While the determination of effective nonlinear optical coefficients in semiconductors for intensities ranging from 0.1–100 TW/cm^2 is especially important for internal ultrafast laser writing, such realistic nonlinear coefficients are also paramount for other applications. In this Section, we give examples of such applications.

Backside processing. Locating energy deposition at the exit surface of semiconductors is essential for optimizing ultrafast laser selective backside ablation and laser lift-off [S94, S95], as well as through-semiconductor ultrafast laser welding [S92, S96]. For both these applications, a misestimation of the critical power for nonlinearities will lead to energy deposition far from the exit surface, and a misestimation of the multi-photon absorption coefficient will lead to either no laser effect, or excessive damage.

Microelectronics security. Ultrashort laser pulses have emerged as a powerful tool for the physical hacking of microelectronic devices. In particular, multi-photon absorption near the gate region of

a transistor can generate free carriers, potentially altering its logical state [S97–S112]. The effective critical power for nonlinearities and the multi-photon absorption coefficient are key parameters governing the spatial localization and density of laser-induced carriers. Accurate knowledge and control of these nonlinear optical coefficients are therefore essential for the development of robust counter-measures against laser-based fault injection or tampering attacks in microelectronic systems.

THz wave generation. The interaction of ultrashort laser pulses with the bulk of semiconductors can lead to the generation of THz radiation via second-order nonlinear processes such as optical rectification. Various semiconductors have been employed for this purpose, including ZnTe, GaAs, CdTe, ZnSe, $ZnGeP_2$, $CdGeP_2$, $CdSiP_2$, and $LiNbO_3$ [S113–S122]. Accurate determination of the critical power for nonlinearities and the multi-photon absorption coefficient is essential for predicting the evolution of the pump pulse inside the material and maintaining phase-matching conditions. Inaccurate estimations can lead to beam distortion, free-carrier generation, and dispersion, all of which degrade THz generation efficiency. Reliable modeling of these nonlinear effects is thus a prerequisite for optimizing the THz yield.

High-harmonic generation (HHG). At the opposite end of the electromagnetic spectrum, semiconductors have emerged as excellent media for HHG, a process that underpins attosecond science. By focusing ultrashort laser pulses into the bulk of materials such as ZnO, GaSe, and Si, HHG has been demonstrated in various solid-state systems [S123–S127]. In this regime, nonlinear refraction and multi-photon absorption play key roles in determining the spatial localization, cutoff energy, yield, and phase-matching conditions of the emitted harmonics. Accurate knowledge of the critical power for nonlinearities and the multi-photon absorption coefficient is therefore crucial for controlling and optimizing HHG in bulk semiconductors.

Supercontinuum generation (SCG). The high nonlinear refractive indices of semiconductors can also be exploited for supercontinuum generation. Multi-octave SCG has been demonstrated in bulk materials such as Si, InP, GaAs, GaN, ZnSe, $ZnGeP_2$ and $LiInS_2$ [S128–S136]. Nonlinear refraction and absorption coefficients are critical, as these parameters govern key processes such as self-phase modulation, pulse splitting, and soliton formation. Their precise values are therefore indispensable for modeling nonlinear propagation dynamics and for reliably predicting the resulting supercontinuum bandwidth and spectral shape.

To qualitatively assess the impact of pulse duration on supercontinuum generation (SCG) efficiency, we performed transverse spectral measurements in the plasma region, as illustrated in Supplementary Fig. 17(a). These measurements consist of transversely collecting with an objective lens (Mitutoyo, M Plan Apo NIR 20×, NA = 0.40) light emitted and scattered by the plasma in Si, before accurate spectral characterizations with an optical spectrum analyzer (OSA, Yokogawa, model AQ6375, wavelength range: 1200–2400 nm). Various input pulse energies $E_{\rm in}$ and pulse durations τ have been used. The corresponding results are shown in Supplementary Fig. 17(b). For long pulse duration ($\tau = 10$ ps), the measured spectrum is nearly identical as the input laser spectrum (see Supplementary Fig. 8(a)). While SCG in Si has previously been reported only for pulse durations $\tau \leq 320$ fs [S131, S133, S136], our results show that spectral broadening—primarily governed by self-phase modulation—also occurs for longer pulses. Noteworthy, when spectral broadening occurs, the blue-shifted sideband is larger than its red counterpart. This asymmetric spectral broadening is a hallmark of free-carrier effects, which induce a dynamic refractive index decrease, and preferentially absorb and scatter long wavelengths, resulting in enhanced blue-shifted emission and suppressed red-side spectral extension.

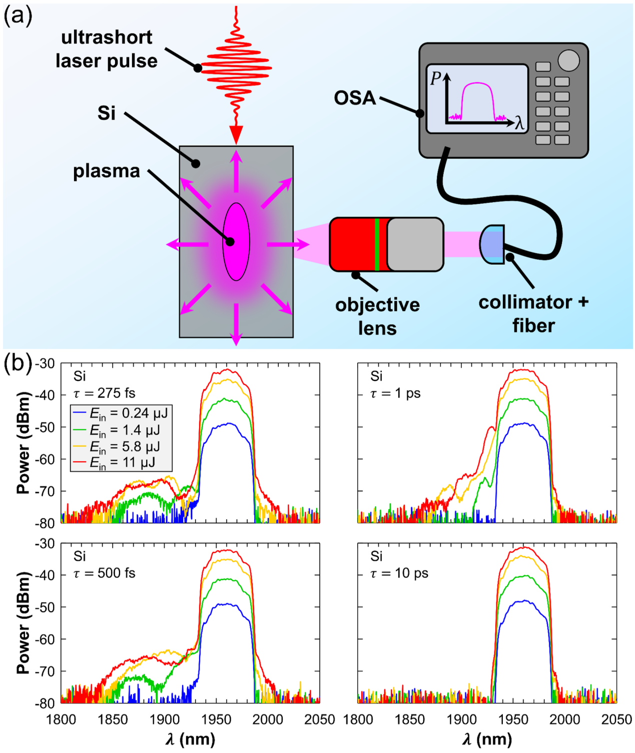

Supplementary Fig. 17 Self-phase modulation in Si. (a) Schematic of the experimental setup used for transverse spectral measurements of light scattered and emitted by the plasma. (b) Spectra detected for different pulse energies $E_{\rm in}$ and pulse durations τ.

References

[S1] Varshni, Y. Temperature dependence of the energy gap in semiconductors. *Physica* **34**, 149–154 (1967). URL https://doi.org/10.1016/0031-8914(67)90062-6.

[S2] Li, H. H. Refractive index of silicon and germanium and its wavelength and temperature derivatives. *Journal of Physical and Chemical Reference Data* **9**, 561–658 (1980). URL https://doi.org/10.1063/1.555624.

[S3] De Leonardis, F., Troia, B., Soref, R. A. & Passaro, V. M. N. Dispersion of nonresonant third-order nonlinearities in GeSiSn ternary alloys. *Scientific Reports* **6**, 32622 (2016). URL https://doi.org/10.1038/srep32622.

[S4] Sohn, B.-U., Monmeyran, C., Kimerling, L. C., Agarwal, A. M. & Tan, D. T. H. Kerr nonlinearity and multi-photon absorption in germanium at mid-infrared wavelengths. *Applied Physics Letters* **111**, 091902 (2017). URL https://doi.org/10.1063/1.4990590.

[S5] Bristow, A. D., Rotenberg, N. & van Driel, H. M. Two-photon absorption and Kerr coefficients of silicon for 850–2200nm. *Applied Physics Letters* **90**, 191104 (2007). URL https://doi.org/10.1063/1.2737359.

[S6] Lin, Q. *et al.* Dispersion of silicon nonlinearities in the near infrared region. *Applied Physics Letters* **91**, 021111 (2007). URL https://doi.org/10.1063/1.2750523.

[S7] Zlatanovic, S. *et al.* Mid-infrared wavelength conversion in silicon waveguides using ultracompact telecom-band-derived pump source. *Nature Photonics* **4**, 561–564 (2010). URL https://doi.org/10.1038/nphoton.2010.117.

[S8] Wang, T. *et al.* Multi-photon absorption and third-order nonlinearity in silicon at mid-infrared wavelengths. *Optics Express* **21**, 32192 (2013). URL https://doi.org/10.1364/OE.21.032192.

[S9] Zhang, L., Agarwal, A. M., Kimerling, L. C. & Michel, J. Nonlinear Group IV photonics based on silicon and germanium: from near-infrared to mid-infrared. *Nanophotonics* **3**, 247–268 (2014). URL https://doi.org/10.1515/nanoph-2013-0020.

[S10] Ensley, T. R. & Bambha, N. K. Ultrafast nonlinear refraction measurements of infrared transmitting materials in the mid-wave infrared. *Optics Express* **27**, 37940 (2019). URL https://doi.org/10.1364/OE.380702.

[S11] Jansonas, G., Budriūnas, R., Vengris, M. & Varanavičius, A. Interferometric measurements of nonlinear refractive index in the infrared spectral range. *Optics Express* **30**, 30507 (2022). URL https://doi.org/10.1364/OE.458850.

[S12] Beaudoin, M., DeVries, A. J. G., Johnson, S. R., Laman, H. & Tiedje, T. Optical absorption edge of semi-insulating GaAs and InP at high temperatures. *Applied Physics Letters* **70**, 3540–3542 (1997). URL https://doi.org/10.1063/1.119226.

[S13] Pettit, G. D. & Turner, W. J. Refractive Index of InP. *Journal of Applied Physics* **36**, 2081–2081 (1965). URL https://doi.org/10.1063/1.1714410.

[S14] Ching, W. Y. & Huang, M.-Z. Calculation of optical excitations in cubic semiconductors. III. Third-harmonic generation. *Physical Review B* **47**, 9479–9491 (1993). URL https://doi.org/10.1103/PhysRevB.47.9479.

[S15] Yu, Y. *et al.* Switching characteristics of an InP photonic crystal nanocavity: Experiment and theory. *Optics Express* **21**, 31047 (2013). URL https://doi.org/10.1364/OE.21.031047.

[S16] Heuck, M. *et al.* Heterodyne pump probe measurements of nonlinear dynamics in an indium phosphide photonic crystal cavity. *Applied Physics Letters* **103**, 181120 (2013). URL https://doi.org/10.1063/1.4828355.

[S17] Jiao, Y. *et al.* Indium Phosphide Membrane Nanophotonic Integrated Circuits on Silicon. *physica status solidi (a)* **217**, 1900606 (2020). URL https://doi.org/10.1002/pssa.201900606.

[S18] Skauli, T. *et al.* Improved dispersion relations for GaAs and applications to nonlinear optics. *Journal of Applied Physics* **94**, 6447–6455 (2003). URL https://doi.org/10.1063/1.1621740.

[S19] Dinu, M., Quochi, F. & Garcia, H. Third-order nonlinearities in silicon at telecom wavelengths. *Applied Physics Letters* **82**, 2954–2956 (2003). URL https://doi.org/10.1063/1.1571665.

[S20] Hurlbut, W. C., Lee, Y.-S., Vodopyanov, K. L., Kuo, P. S. & Fejer, M. M. Multiphoton absorption and nonlinear refraction of GaAs in the mid-infrared. *Optics Letters* **32**, 668 (2007). URL https://doi.org/10.1364/OL.32.000668.

[S21] Zha, C. *et al.* Optical properties and structural correlations of GeAsSe chalcogenide glasses. *Journal of Materials Science: Materials in Electronics* **18**, 389–392 (2007). URL https://doi.org/10.1007/s10854-007-9235-3.

[S22] Joërg, A., Lemarchand, F., Zhang, M., Lequime, M. & Lumeau, J. Optical characterization of photosensitive AMTIR-1 chalcogenide thin layers deposited by electron beam deposition. *Journal of Non-Crystalline Solids* **442**, 22–28 (2016). URL https://doi.org/10.1016/j.jnoncrysol.2016.03.018.

[S23] Miller, A. & Clark, W. Electrical Properties of $ZnGeP_2$ and $CdGeP_2$. *Le Journal de Physique Colloques* **36**, C3–73–C3–75 (1975). URL https://doi.org/10.1051/jphyscol:1975314.

[S24] Boyd, G. D., Buehler, E. & Storz, F. G. Linear and nonlinear optical properties of $ZnGeP_2$ and CdSe. *Applied Physics Letters* **18**, 301–304 (1971). URL https://doi.org/10.1063/1.1653673.

[S25] Patel, A., Singh, D., Sonvane, Y., Thakor, P. & Ahuja, R. Bulk and monolayer As_2S_3 as promising thermoelectric material with high conversion performance. *Computational Materials Science* **183**, 109913 (2020). URL https://doi.org/10.1016/j.commatsci.2020.109913.

[S26] Rodney, W. S., Malitson, I. H. & King, T. A. Refractive Index of Arsenic Trisulfide. *Journal of the Optical Society of America* **48**, 633 (1958). URL https://doi.org/10.1364/JOSA.48.000633.

[S27] McCanny, J. V. & Murray, R. B. The band structures of gallium and indium selenide. *Journal of Physics C: Solid State Physics* **10**, 1211–1222 (1977). URL https://doi.org/10.1088/0022-3719/10/8/022.

[S28] Kato, K., Tanno, F. & Umemura, N. Sellmeier and thermo-optic dispersion formulas for GaSe (Revisited). *Applied Optics* **52**, 2325 (2013). URL https://doi.org/10.1364/AO.52.002325.

[S29] Kittel, C. *Introduction to solid state physics* (John Wiley & Sons, Inc., 2005). URL https://doi.org/10.1016/0022-5096(57)90051-0.

[S30] Bond, W. L. Measurement of the Refractive Indices of Several Crystals. *Journal of Applied Physics* **36**, 1674–1677 (1965). URL https://doi.org/10.1063/1.1703106.

[S31] Kawamori, T., Schunemann, P. G., Gruzdev, V. & Vodopyanov, K. L. High-order (N = 4–6) multiphoton absorption and mid-infrared Kerr nonlinearity in GaP, ZnSe, GaSe, and ZGP crystals. *APL Photonics* **7**, 086101 (2022). URL https://doi.org/10.1063/5.0089925.

[S32] Isik, M., Gullu, H., Parlak, M. & Gasanly, N. Synthesis and temperature-tuned band gap characteristics of magnetron sputtered ZnTe thin films. *Physica B: Condensed Matter* **582**, 411968 (2020). URL https://doi.org/10.1016/j.physb.2019.411968.

[S33] Li, H. H. Refractive Index of ZnS, ZnSe, and ZnTe and Its Wavelength and Temperature Derivatives. *Journal of Physical and Chemical Reference Data* **13**, 103–150 (1984). URL https://doi.org/10.1063/1.555705.

[S34] Itoh, N., Fujinaga, T. & Nakau, T. Birefringence in $CdSiP_2$. *Japanese Journal of Applied Physics* **17**, 951–952 (1978). URL https://doi.org/10.1143/JJAP.17.951.

[S35] Wei, J. *et al.* Measurement of refractive indices of $CdSiP_2$ at temperatures from 90 to 450 K. *Optical Materials Express* **8**, 235 (2018). URL https://doi.org/10.1364/OME.8.000235.

[S36] Ferdinandus, M. R. *et al.* Nonlinear optical measurements of $CdSiP_2$ at near and mid-infrared wavelengths. *Optical Materials Express* **10**, 2066 (2020). URL https://doi.org/10.1364/OME.399516.

[S37] Marcinkevičiūtė, A., Tamošauskas, G. & Dubietis, A. Supercontinuum generation in mixed thallous halides KRS-5 and KRS-6. *Optical Materials* **78**, 339–344 (2018). URL https://doi.org/10.1016/j.optmat.2018.02.042.

[S38] Rodney, W. S. & Malitson, I. H. Refraction and Dispersion of Thallium Bromide Iodide. *Journal of the Optical Society of America* **46**, 956 (1956). URL https://doi.org/10.1364/JOSA.46.000956.

[S39] Chen, S., Gong, X. G. & Wei, S.-H. Band-structure anomalies of the chalcopyrite semiconductors $CuGaX_2$ versus $AgGaX_2$ (X=S and Se) and their alloys. *Physical Review B* **75**, 205209 (2007). URL https://doi.org/10.1103/PhysRevB.75.205209.

[S40] Takaoka, E. & Kato, K. Thermo-optic dispersion formula for $AgGaS_2$. *Applied Optics* **38**, 4577 (1999). URL https://doi.org/10.1364/AO.38.004577.

[S41] Streetman, B. G., Banerjee, S. K. *Solid State Electronic Devices* (Pearson Education Limited, 2016). URL https://doi.org/10.1088/0031-9112/24/3/014.

[S42] Marple, D. T. F. Refractive Index of ZnSe, ZnTe, and CdTe. *Journal of Applied Physics* **35**, 539–542 (1964). URL https://doi.org/10.1063/1.1713411.

[S43] Yelisseyev, A., Lobanov, S., Krinitsin, P. & Isaenko, L. The optical properties of the nonlinear crystal $BaGa_4Se_7$. *Optical Materials* **99**, 109564 (2020). URL https://doi.org/10.1016/j.optmat.2019.109564.

[S44] Zhai, N. *et al.* Temperature-Dependent Sellmeier Equations of IR Nonlinear Optical Crystal $BaGa_4Se_7$. *Crystals* **7**, 62 (2017). URL https://doi.org/10.3390/cryst7030062.

[S45] Hettner, G. & Leisegang, G. Die Dispersion der Mischkristalle TlBr-TlJ (KRS5) und TlCl-TlBr (KRS6) im Ultrarot. *Optik* **3**, 305–314 (1948).

[S46] Klimm, D. Electronic materials with a wide band gap: recent developments. *IUCrJ* **1**, 281–290 (2014). URL https://doi.org/10.1107/S2052252514017229.

[S47] Wang, S. *et al.* 4H-SiC: a new nonlinear material for midinfrared lasers. *Laser & Photonics Reviews* **7**, 831–838 (2013). URL https://doi.org/10.1002/lpor.201300068.

[S48] Cardenas, J. *et al.* Optical nonlinearities in high-confinement silicon carbide waveguides. *Optics Letters* **40**, 4138 (2015). URL https://doi.org/10.1364/OL.40.004138.

[S49] Barker, A. S. & Ilegems, M. Infrared Lattice Vibrations and Free-Electron Dispersion in GaN. *Physical Review B* **7**, 743–750 (1973). URL https://doi.org/10.1103/PhysRevB.7.743.

[S50] Almeida, G. F. B. *et al.* Third-Order Nonlinear Spectrum of GaN under Femtosecond-Pulse Excitation from the Visible to the Near Infrared. *Photonics* **6**, 69 (2019). URL https://doi.org/10.3390/photonics6020069.

[S51] Debenham, M. Refractive indices of zinc sulfide in the 0.405–13-μm wavelength range. *Applied Optics* **23**, 2238 (1984). URL https://doi.org/10.1364/AO.23.002238.

[S52] Yelisseyev, A., Lin, Z. S., Starikova, M., Isaenko, L. & Lobanov, S. Optical transitions due to native defects in nonlinear optical crystals $LiGaS_2$. *Journal of Applied Physics* **111**, 113507 (2012). URL https://doi.org/10.1063/1.4723645.

[S53] Vu, T. V. *et al.* Optical and electronic properties of lithium thiogallate ($LiGaS_2$): experiment and theory. *RSC Advances* **10**, 26843–26852 (2020). URL https://doi.org/10.1039/D0RA03280H.

[S54] Singh, P., Harbola, M. K. & Johnson, D. D. Better band gaps for wide-gap semiconductors from a locally corrected exchange-correlation potential that nearly eliminates self-interaction errors. *Journal of Physics: Condensed Matter* **29**, 424001 (2017). URL https://doi.org/10.1088/1361-648X/aa837b. 1704.06245.

[S55] Luke, K., Okawachi, Y., Lamont, M. R. E., Gaeta, A. L. & Lipson, M. Broadband mid-infrared frequency comb generation in a Si_3N_4 microresonator. *Optics Letters* **40**, 4823 (2015). URL https://doi.org/10.1364/OL.40.004823.

[S56] Ikeda, K., Saperstein, R. E., Alic, N. & Fainman, Y. Thermal and Kerr nonlinear properties of plasma-deposited silicon nitride/ silicon dioxide waveguides. *Optics Express* **16**, 12987 (2008). URL https://doi.org/10.1364/OE.16.012987.

[S57] Neufeld, S., Schindlmayr, A. & Schmidt, W. G. Quasiparticle energies and optical response of $RbTiOPO_4$ and $KTiOAsO_4$. *Journal of Physics: Materials* **5**, 015002 (2022). URL https://doi.org/10.1088/2515-7639/ac3384.

[S58] Cheng, L. K., Cheng, L.-T., Bierlein, J. D., Zumsteg, F. C. & Ballman, A. A. Properties of doped and undoped crystals of single domain $KTiOAsO_4$. *Applied Physics Letters* **62**, 346–348 (1993). URL https://doi.org/10.1063/1.108953.

[S59] Phillip, H. R. & Taft, E. A. Kramers-Kronig Analysis of Reflectance Data for Diamond. *Physical Review* **136**, A1445–A1448 (1964). URL https://doi.org/10.1103/PhysRev.136.A1445.

[S60] Hausmann, B. J. M., Bulu, I., Venkataraman, V., Deotare, P. & Lončar, M. Diamond nonlinear photonics. *Nature Photonics* **8**, 369–374 (2014). URL https://doi.org/10.1038/nphoton.2014.72.

[S61] Pastrňák, J. & Roskovcová, L. Refraction Index Measurements on AlN Single Crystals. *physica status solidi (b)* **14**, K5–K8 (1966). URL https://doi.org/10.1002/pssb.19660140127.

[S62] Jung, H., Xiong, C., Fong, K. Y., Zhang, X. & Tang, H. X. Optical frequency comb generation from aluminum nitride microring resonator. *Optics Letters* **38**, 2810 (2013). URL https://doi.org/10.1364/OL.38.002810.

[S63] Malitson, I. H. Refraction and Dispersion of Synthetic Sapphire. *Journal of the Optical Society of America* **52**, 1377 (1962). URL https://doi.org/10.1364/JOSA.52.001377.

[S64] Patwardhan, G. N., Ginsberg, J. S., Chen, C. Y., Jadidi, M. M. & Gaeta, A. L. Nonlinear refractive index of solids in mid-infrared. *Optics Letters* **46**, 1824 (2021). URL https://doi.org/10.1364/OL.421469.

[S65] Fischetti, M. V., DiMaria, D. J., Brorson, S. D., Theis, T. N. & Kirtley, J. R. Theory of high-field electron transport in silicon dioxide. *Physical Review B* **31**, 8124–8142 (1985). URL https://doi.org/10.1103/PhysRevB.31.8124.

[S66] Malitson, I. H. Interspecimen Comparison of the Refractive Index of Fused Silica. *Journal of the Optical Society of America* **55**, 1205 (1965). URL https://doi.org/10.1364/JOSA.55.001205.

[S67] Tsujibayashi, T., Toyoda, K., Sakuragi, S., Kamada, M. & Itoh, M. Spectral profile of the two-photon absorption coefficients in CaF_2 and BaF_2. *Applied Physics Letters* **80**, 2883–2885 (2002). URL https://doi.org/10.1063/1.1471939.

[S68] Li, H. H. Refractive index of alkaline earth halides and its wavelength and temperature derivatives. *Journal of Physical and Chemical Reference Data* **9**, 161–290 (1980). URL https://doi.org/10.1063/1.555616.

[S69] Chaney, R. C., Lafon, E. E. & Lin, C. C. Energy Band Structure of Lithium Fluoride Crystals by the Method of Tight Binding. *Physical Review B* **4**, 2734–2741 (1971). URL https://doi.org/10.1103/PhysRevB.4.2734.

[S70] Li, H. H. Refractive index of alkali halides and its wavelength and temperature derivatives. *Journal of Physical and Chemical Reference Data* **5**, 329–528 (1976). URL https://doi.org/10.1063/1.555536.

[S71] Thomas, J., Stephan, G., Lemonnier, J. C., Nisar, M. & Robin, S. Optical Anisotropy of MgF, in Its UV Absorption Region. *Physica Status Solidi (b)* **56**, 163–170 (1973). URL https://doi.org/10.1002/pssb.2220560115.

[S72] Fibich, G. & Gaeta, A. L. Critical power for self-focusing in bulk media and in hollow waveguides. *Optics Letters* **25**, 335 (2000). URL https://doi.org/10.1364/ol.25.000335.

[S73] Hon, N. K., Soref, R. & Jalali, B. The third-order nonlinear optical coefficients of Si, Ge, and $Si_{1-x}Ge_x$ in the midwave and longwave infrared. *Journal of Applied Physics* **110**, 011301 (2011). URL https://doi.org/10.1063/1.3592270.

[S74] Garcia, H. & Avanaki, K. N. Direct and indirect two-photon absorption in Ge within the effective mass approximation. *Applied Physics Letters* **100**, 131105 (2012). URL https://doi.org/10.1063/1.3693389.

[S75] Mashanovich, G. Z. *et al.* Germanium Mid-Infrared Photonic Devices. *Journal of Lightwave Technology* **35**, 624–630 (2017). URL https://doi.org/10.1109/JLT.2016.2632301.

[S76] Euser, T. G. & Vos, W. L. Spatial homogeneity of optically switched semiconductor photonic crystals and of bulk semiconductors. *Journal of Applied Physics* **97**, 043102 (2005). URL https://doi.org/10.1063/1.1846949.

[S77] Tiedje, H., Haugen, H. & Preston, J. Measurement of nonlinear absorption coefficients in GaAs, InP and Si by an optical pump THz probe technique. *Optics Communications* **274**, 187–197 (2007). URL https://doi.org/10.1016/j.optcom.2007.01.049.

[S78] Benis, S. *et al.* Three-photon absorption spectra and bandgap scaling in direct-gap semiconductors. *Optica* **7**, 888 (2020). URL https://doi.org/10.1364/OPTICA.396056. 2005.06077.

[S79] Wherrett, B. S. Scaling rules for multiphoton interband absorption in semiconductors. *Journal of the Optical Society of America B* **1**, 67 (1984). URL https://doi.org/10.1364/JOSAB.1.000067.

[S80] Peceli, D. *et al.* Three-Photon Absorption of GaAs and other Semiconductors. In *Nonlinear Optics*, NTu1B.6 (OSA, Washington, D.C., 2013). URL https://doi.org/10.1364/NLO.2013.NTu1B.6.

[S81] De Kernier, I. *et al.* C-RED 2 ER: an extended range SWIR camera with applications in hyperspectral imaging. In *Proceedings of SPIE*, 119970V (SPIE, 2022). URL https://doi.org/10.1117/12.2624018.

[S82] Mareev, E. I. *et al.* Effect of pulse duration on the energy delivery under nonlinear propagation of tightly focused Cr:forsterite laser radiation in bulk silicon. *Laser Physics Letters* **17**, 015402 (2020). URL https://doi.org/10.1088/1612-202X/ab5d23.

[S83] Bourgeade, A., Donval, T., Gallais, L., Lamaignère, L. & Rullier, J.-L. Modeling surface defects in fused silica optics for laser wave propagation. *Journal of the Optical Society of America B* **32**, 655 (2015). URL https://doi.org/10.1364/JOSAB.32.000655.

[S84] Chambonneau, M. *et al.* Investigations on laser damage growth in fused silica with simultaneous wavelength irradiation. *Applied Optics* **54**, 1463 (2015). URL https://doi.org/10.1364/AO.54.001463.

[S85] Chambonneau, M. *et al.* Transverse ultrafast laser inscription in bulk silicon. *Physical Review Research* **3**, 043037 (2021). URL https://doi.org/10.1103/PhysRevResearch.3.043037. 2104.12084.

[S86] Wang, A., Das, A. & Grojo, D. Ultrafast Laser Writing Deep inside Silicon with THz-Repetition-Rate Trains of Pulses. *Research* **2020**, 1–11 (2020). URL https://doi.org/10.34133/2020/8149764.

[S87] Li, Q., Chambonneau, M., Blothe, M., Gross, H. & Nolte, S. Flexible, fast, and benchmarked vectorial model for focused laser beams. *Applied Optics* **60**, 3954 (2021). URL https://doi.org/10.1364/AO.421945.

[S88] Li, Q. InFocus (2021). URL https://github.com/QF06/InFocus.

[S89] Chanal, M. *et al.* Crossing the threshold of ultrafast laser writing in bulk silicon. *Nature Communications* **8**, 773 (2017). URL https://doi.org/10.1038/s41467-017-00907-8.

[S90] Salzberg, C. D. & Villa, J. J. Infrared Refractive Indexes of Silicon Germanium and Modified Selenium Glass. *Journal of the Optical Society of America* **47**, 244 (1957). URL https://doi.org/10.1364/JOSA.47.000244.

[S91] Burnett, J. H., Kaplan, S. G., Stover, E. & Phenis, A. Refractive index measurements of Ge. In LeVan, P. D., Sood, A. K., Wijewarnasuriya, P. & D'Souza, A. I. (eds.) *Infrared Sensors, Devices, and Applications VI*, vol. 9974, 99740X (2016). URL https://doi.org/10.1117/12.2237978.

[S92] Chambonneau, M. *et al.* Taming Ultrafast Laser Filaments for Optimized Semiconductor–Metal Welding. *Laser & Photonics Reviews* **15**, 2000433 (2021). URL https://doi.org/10.1002/lpor.202000433.

[S93] Marburger, J. Self-focusing: theory. *Progress in Quantum Electronics* **4**, 35–110 (1975). URL https://doi.org/10.1109/IQEC.2005.1561127.

[S94] Lei, S., Grojo, D., Ma, J., Yu, X. & Wu, H. Femtosecond Laser Backside Ablation of Gold Film on Silicon Substrate. *Procedia Manufacturing* **5**, 594–608 (2016). URL https://doi.org/10.1016/j.promfg.2016.08.049.

[S95] Astrauskas, I., Považay, B., Baltuška, A. & Pugžlys, A. Influence of 2.09-μm pulse duration on through-silicon laser ablation of thin metal coatings. *Optics & Laser Technology Laser Technology* **133**, 106535 (2021). URL https://doi.org/10.1016/j.optlastec.2020.106535.

[S96] Chambonneau, M., Li, Q., Blothe, M., Arumugam, S. V. & Nolte, S. Ultrafast Laser Welding of Silicon. *Advanced Photonics Research* **4**, 2200300 (2023). URL https://doi.org/10.1002/adpr.202200300. 2211.03518.

[S97] McMorrow, D., Lotshaw, W., Melinger, J., Buchner, S. & Pease, R. Subbandgap laser-induced single event effects: carrier generation via two-photon absorption. *IEEE Transactions on Nuclear Science* **49**, 3002–3008 (2002). URL https://doi.org/10.1109/TNS.2002.805337.

[S98] El-Mamouni, F. *et al.* Laser- and Heavy Ion-Induced Charge Collection in Bulk FinFETs. *IEEE Transactions on Nuclear Science* **58**, 2563–2569 (2011). URL https://doi.org/10.1109/TNS.2011.2171994.

[S99] Khachatrian, A. *et al.* A Dosimetry Methodology for Two-Photon Absorption Induced Single-Event Effects Measurements. *IEEE Transactions on Nuclear Science* **61**, 3416–3423 (2014). URL https://doi.org/10.1109/TNS.2014.2369006.

[S100] Hales, J. M. *et al.* Simulation of Laser-Based Two-Photon Absorption Induced Charge Carrier Generation in Silicon. *IEEE Transactions on Nuclear Science* **62**, 1550–1557 (2015). URL https://doi.org/10.1109/TNS.2015.2422793.

[S101] Della Marca, V. *et al.* NVM cell degradation induced by femtosecond laser backside irradiation for reliability tests. In *2016 IEEE International Reliability Physics Symposium (IRPS)*, vol. 2016-Septe, 7B–4–1–7B–4–7 (IEEE, 2016). URL https://doi.org/10.1109/IRPS.2016.7574580.

[S102] Chambonneau, M. *et al.* Suppressing the memory state of floating gate transistors with repeated femtosecond laser backside irradiations. *Applied Physics Letters* **110**, 6 (2017). URL https://doi.org/10.1063/1.4982032.

[S103] Hales, J. M. *et al.* Strong Correlation Between Experiment and Simulation for Two-Photon Absorption Induced Carrier Generation. *IEEE Transactions on Nuclear Science* **64**, 1133–1136 (2017). URL https://doi.org/10.1109/TNS.2017.2686010.

[S104] Hales, J. M. *et al.* Experimental Validation of an Equivalent LET Approach for Correlating Heavy-Ion and Laser-Induced Charge Deposition. *IEEE Transactions on Nuclear Science* **65**, 1724–1733 (2018). URL https://doi.org/10.1109/TNS.2018.2828332.

[S105] Ildefonso, A. *et al.* Optimizing Optical Parameters to Facilitate Correlation of Laser- and Heavy-Ion-Induced Single-Event Transients in SiGe HBTs. *IEEE Transactions on Nuclear Science* **66**, 359–367 (2019). URL https://doi.org/10.1109/TNS.2018.2882821.

[S106] Chiquet, P. *et al.* Phenomenological modelling of non-volatile memory threshold voltage shift induced by nonlinear ionization with a femtosecond laser. *Scientific Reports* **9**, 7392 (2019). URL https://doi.org/10.1038/s41598-019-43344-x.

[S107] Hales, J. M. *et al.* Using Bessel beams and two-photon absorption to predict radiation effects in microelectronics. *Optics Express* **27**, 37652 (2019). URL https://doi.org/10.1364/OE.27.037652.

[S108] Hales, J. M. *et al.* New Approach for Pulsed-Laser Testing That Mimics Heavy-Ion Charge Deposition Profiles. *IEEE Transactions on Nuclear Science* **67**, 81–90 (2020). URL https://doi.org/10.1109/TNS.2019.2950431.

[S109] Ryder, L. D. *et al.* Polarization Dependence of Pulsed Laser-Induced SEEs in SOI FinFETs. *IEEE Transactions on Nuclear Science* **67**, 38–43 (2020). URL https://doi.org/10.1109/TNS.2019.2956911.

[S110] Ryder, L. D. *et al.* Simulation of Pulsed-Laser-Induced Testing in Microelectronic Devices. *IEEE Transactions on Nuclear Science* **68**, 2496–2507 (2021). URL https://doi.org/10.1109/TNS.2021.3111864.

[S111] Hales, J. M. *et al.* Pulsed-Laser Testing to Quantitatively Evaluate Latchup Sensitivity in Mixed-Signal ASICs. *IEEE Transactions on Nuclear Science* **69**, 429–435 (2022). URL https://doi.org/10.1109/TNS.2021.3129416.

[S112] Hales, J. M. *et al.* Quantitative Prediction of Ion-Induced Single-Event Transients in an Operational Amplifier Using a Quasi-Bessel Beam Pulsed-Laser Approach. *IEEE Transactions on Nuclear Science* **70**, 354–362 (2023). URL https://doi.org/10.1109/TNS.2022.3232724.

[S113] Aggarwal, R. L., Lax, B. & Favrot, G. Noncollinear phase matching in GaAs. *Applied Physics Letters* **22**, 329–330 (1973). URL https://doi.org/10.1063/1.1654658.

[S114] Hebling, J., Almasi, G., Kozma, I. & Kuhl, J. Velocity matching by pulse front tilting for large area THz-pulse generation. *Optics Express* **10**, 1161 (2002). URL https://doi.org/10.1364/oe.10.001161.

[S115] Blanchard, F. *et al.* Generation of 1.5 μJ single-cycle terahertz pulses by optical rectification from a large aperture ZnTe crystal. *Optics Express* **15**, 13212 (2007). URL https://doi.org/10.1364/OE.15.013212.

[S116] Fülöp, J. A., Pálfalvi, L., Almási, G. & Hebling, J. Design of high-energy terahertz sources based on optical rectification. *Optics Express* **18**, 12311 (2010). URL https://doi.org/10.1364/OE.18.012311.

[S117] Hirori, H., Doi, A., Blanchard, F. & Tanaka, K. Single-cycle terahertz pulses with amplitudes exceeding 1 MV/cm generated by optical rectification in $LiNbO_3$. *Applied Physics Letters* **98**, 2–5 (2011). URL https://doi.org/10.1063/1.3560062.

[S118] Bakunov, M. I., Bodrov, S. B. & Mashkovich, E. A. Terahertz generation with tilted-front laser pulses: dynamic theory for low-absorbing crystals. *Journal of the Optical Society of America B* **28**, 1724 (2011). URL https://doi.org/10.1364/josab.28.001724.

[S119] Fülöp, J. A. *et al.* Efficient generation of THz pulses with 04 mJ energy. *Optics Express* **22**, 20155 (2014). URL https://doi.org/10.1364/OE.22.020155. arXiv:1109.6852.

[S120] Bakunov, M. I. & Bodrov, S. B. Terahertz generation with tilted-front laser pulses in a contact-grating scheme. *Journal of the Optical Society of America B* **31**, 2549 (2014). URL https://doi.org/10.1364/josab.31.002549.

[S121] Blanchard, F. *et al.* Terahertz pulse generation from bulk GaAs by a tilted-pulse-front excitation at 1.8 μm. *Applied Physics Letters* **105**, 3–7 (2014). URL https://doi.org/10.1063/1.4904005.

[S122] Piyathilaka, H. P. *et al.* Terahertz generation by optical rectification in chalcopyrite crystals $ZnGeP_2$, $CdGeP_2$ and $CdSiP_2$. *Optics Express* **27**, 16958 (2019). URL https://doi.org/10.1364/OE.27.016958.

[S123] Ghimire, S. *et al.* Observation of high-order harmonic generation in a bulk crystal. *Nature Physics* **7**, 138–141 (2011). URL https://doi.org/10.1038/nphys1847.

[S124] Schubert, O. *et al.* Sub-cycle control of terahertz high-harmonic generation by dynamical Bloch oscillations. *Nature Photonics* **8**, 119–123 (2014). URL https://doi.org/10.1038/nphoton.2013.349.

[S125] Vampa, G. *et al.* Linking high harmonics from gases and solids. *Nature* **522**, 462–464 (2015). URL https://doi.org/10.1038/nature14517.

[S126] Vampa, G. *et al.* Plasmon-enhanced high-harmonic generation from silicon. *Nature Physics* **13**, 659–662 (2017). URL https://doi.org/10.1038/nphys4087.

[S127] Goulielmakis, E. & Brabec, T. High harmonic generation in condensed matter. *Nature Photonics* **16**, 411–421 (2022). URL https://doi.org/10.1038/s41566-022-00988-y.

[S128] Zhou, B. & Bache, M. Invited Article: Multiple-octave spanning high-energy mid-IR supercontinuum generation in bulk quadratic nonlinear crystals. *APL Photonics* **1** (2016). URL https://doi.org/10.1063/1.4953177.

[S129] Mouawad, O. *et al.* Filament-induced visible-to-mid-IR supercontinuum in a ZnSe crystal: Towards multi-octave supercontinuum absorption spectroscopy. *Optical Materials* **60**, 355–358 (2016). URL https://doi.org/10.1016/j.optmat.2016.08.009.

[S130] Šuminas, R. *et al.* Multi-octave spanning nonlinear interactions induced by femtosecond filamentation in polycrystalline ZnSe. *Applied Physics Letters* **110**, 1–5 (2017). URL https://doi.org/10.1063/1.4986440.

[S131] Kadan, V. *et al.* Spatio-temporal dynamics of femtosecond laser pulses at 1550 nm wavelength in crystal silicon. *Applied Physics A* **124**, 560 (2018). URL https://doi.org/10.1007/s00339-018-1986-6.

[S132] Werner, K. *et al.* Ultrafast mid-infrared high harmonic and supercontinuum generation with n 2 characterization in zinc selenide. *Optics Express* **27**, 2867 (2019). URL https://doi.org/10.1364/OE.27.002867.

[S133] Marcinkevičiūtė, A. *et al.* Femtosecond filamentation and supercontinuum generation in bulk silicon. *Optics Letters* **44**, 1343 (2019). URL https://doi.org/10.1364/OL.44.001343.

[S134] Šuminas, R., Marcinkevičiūtė, A., Tamošauskas, G. & Dubietis, A. Even and odd harmonics-enhanced supercontinuum generation in zinc-blende semiconductors. *Journal of the Optical Society of America B* **36**, A22 (2019). URL https://doi.org/10.1364/JOSAB.36.000A22.

[S135] Šuminienė, A., Jukna, V., Šuminas, R., Tamošauskas, G. & Dubietis, A. Femtosecond infrared supercontinuum generation in 6H-SiC crystal. *OSA Continuum* **4**, 911 (2021). URL https://doi.org/10.1364/OSAC.419234.

[S136] Danilin, R. *et al.* Supercontinuum generation in oxide and semiconductor materials (InP, Si, GaN, GaAs, $PbMoO_4$, YVO_4 , ZGP, TiO_2 , diamond) pumped by radiation of the Cr:ZnS fs-MOPA system. *Journal of the Optical Society of America B* **42**, 1315 (2025). URL https://doi.org/10.1364/JOSAB.553197.
