# Supplementary material for: Extreme optical nonlinearities unveiled by ultrafast laser filamentation in semiconductors
Source: Nat Commun. 2026 Feb 14;17:1701. doi: 10.1038/s41467-026-69530-w (PMC12909925; doi:10.1038/s41467-026-69530-w)
Supplement: Supplementary file 2 — Description of Additional Supplementary Files [file 41467_2026_69530_MOESM2_ESM.pdf]

Description of Additional Supplementary Files

File Name: Supplementary Video 1
Description: Pulse duration and input pulse energy dependence of the fraction of absorbed energy f_E for all tested semiconductors.

File Name: Supplementary Video 2
Description: Pulse duration and input pulse energy dependence of the characteristic absorption length L_{abs} for all tested semiconductors.
